# Deposit characterizations and engineering-geotechical modeling for sustainable urbanisation in the Mila basin (NE Algeria)

**DOI:** 10.1038/s41598-026-53104-3

**Published:** 2026-05-25

**Authors:** Khellaf Khoudir, Mihoub Redouane, Kraimat Mohamed, Abdelaziz Rabehi, Mustapha Habib, Amal H. Alharbi, El-Sayed M. El-Kenawy

**Affiliations:** 1 Valorization and Conservation of Arid Ecosystems Laboratory, Faculty of Natural, Life and Earth Sciences, University of Ghardaïa, 47000 Bounoura, Algeria; 2 Faculté des Sciences, Département de Biologie, Laboratoire des réservoirs souterrains pétroliers gaziers et aquifers, Université Amar Telidji Laghouat, Université Kasdi Merbah Ouargla, Ouargla, Algeria; 3 Laboratory of Telecommunication and Smart Systems (LTSS), Faculty of Science and Technology, University of Djelfa, PO Box 3117, 17000 Djelfa, Algeria; 4 Civil and Architectural Engineering, KTH Royal Institute of Technology, Teknikringen, 78, Stockholm, 11428 Sweden; 5 Department of Computer Sciences, College of Computer and Information Sciences, Princess Nourah Bint Abdulrahman University, 11671 Riyadh, Saudi Arabia; 6 Department for Communications and Electronics, Delta Higher Institute of Engineering and Technology, Mansoura, 35511 Egypt; 7 Jadara Research Center, Jadara University, Irbid, 21110 Jordan

**Keywords:** Clayey deposits, Urban, Engineering-model, Mila, Algeria, Engineering, Environmental sciences, Natural hazards, Solid Earth sciences

## Abstract

Rapid urban expansion in Mila (Algeria) has increased exposure to geotechnical hazards related to weak clayey deposits, slope instability, and seismic activity. Its southeastern part (Sanaoua) is particularly affected by settlement, landslides… limiting sustainable urban development. This study presents an integrated geotechnical, geophysical, and hydrogeological investigation to assess subsurface conditions and their implications for foundation performance. The investigation program included Fifteen core drillings (CD), Six pressuremeter (Ps), Twenty-five dynamic penetration tests (P), Ten electrical resistivity tomographiy profiles (ERT), and piezometric monitoring at 10 points (Pz), complemented by laboratory testing. CD results indicate variable colluvial deposits (≈ 0.5–3 m) overlying gray clays containing limestone blocks. These clays exhibit high plasticity (Wl ≈ 61–79%, Ip ≈ 23–49%), near-saturated conditions (Sr ≈ 90%), low to moderate undrained shear strength parameters (c ≈ 0.018–0.043 MPa; φ ≈ 9–16°), and low sulfate content. Ps tests reveal very low stiffness and bearing capacity in the upper 2 m (E_m_ ≈ 6 MPa; q_ad_ < 0.15 MPa), with a progressive increase in mechanical competence below 4–6 m and q_ad_ ≈ 0.45 MPa at depths exceeding 6–8 m. Settlement analyses indicate excessive deformations (60 mm) in shallow layers, decreasing to less than 10 mm at depths greater than 6–8 m. P results highlight strong spatial variability and locally very high resistance values related to limestone blocks, with bedrock depths ranging from 4 to 10 m. ERT profiles confirm thick clay-dominated conductive domains (10–40 Ω.m) locally interrupted by resistive limestone bodies (150 Ω m), explaining the pronounced lateral variability in bearing conditions. Pz monitoring indicates the absence of a permanent groundwater within the explored depth during the monitoring period. Based on these findings, a practical engineering-geological model is proposed within a Mohr–Coulomb framework, demonstrating that shallow foundations are generally unsuitable and that deep or improved foundation systems, together with effective surface-water management, are required to support slope stabilization, erosion control, and seismic-resilient urban development. The main limitations are spatial and depth coverage, temporal monitoring constraints, method-specific uncertainties, and simplifications in modeling. Despite these, the study provides a robust framework for guiding foundation design, slope stabilization, and urban development—but local variations and long-term effects should be addressed with further site-specific investigations.

## Introduction

The Mila Basin (northeastern Algeria) is currently undergoing rapid urban expansion driven by sustained demographic growth and increasing socio-economic demands^1^. This development is occurring within a geologically complex environment characterized by highly heterogeneous soil conditions, including clay-rich deposits that are particularly prone to instability^2,3^. These materials are widely associated with differential settlement, slope instability, and infrastructure damage, especially in areas affected by variable moisture conditions and complex topography^4,5^. As a result, several residential zones in Mila have experienced structural cracking and ground deformation, posing significant challenges for urban planning and geotechnical risk management^6,7^.

Urban expansion in the southeastern part of Mila City, particularly in the Sanaoua sector, has intensified these challenges. This area already exhibits pronounced ground deformation and recurrent instability related to unfavorable subsurface conditions^5,8^. At the same time, the increasing demand for new developable land has led to the designation of such areas as priority zones for urban growth^9^. This situation creates a critical conflict between urbanization pressures and the geotechnical constraints imposed by the local geological framework.

Previous studies have investigated the geological and geotechnical characteristics of the Mila Basin. Early work by Coiffait^10^ established a lithostratigraphic framework, identifying alternating clayey, silty, and sandy deposits with localized gypsum and marly horizons. Subsequent hydrogeological studies^8,9^ demonstrated that seasonal groundwater fluctuations significantly influence soil compressibility and shear strength, thereby increasing the susceptibility to settlement and slope instability. Geotechnical investigations have further shown that these clay-rich soils exhibit high plasticity indices and low undrained shear strength, making them particularly sensitive to both static and seismic loading^5,11^. Additional studies^2,3^ documented widespread subsidence and localized landslides, attributing these processes to the combined effects of clay mineralogy, groundwater variations, and basin morphology. Moreover, the presence of gypsum and associated sulfate content has been identified as a contributing factor to volumetric changes and foundation heave^4^. However, these studies remain largely descriptive or regional in scope and generally lack detailed in situ mechanical characterization and integrated analysis of subsurface variability.

At the international scale, similar geotechnical challenges have been widely reported in urban areas developed on clay-rich and heterogeneous deposits, particularly in Mediterranean and semi-arid regions. Numerous studies have demonstrated that high-plasticity clays subjected to variable moisture conditions exhibit significant compressibility, low shear strength, and high susceptibility to deformation and instability. To address these issues, integrated investigation approaches have increasingly been adopted, combining in situ testing methods such as pressuremeter testing and penetration tests with geophysical techniques, notably electrical resistivity tomography (ERT). These methods enable improved characterization of subsurface heterogeneity and the identification of mechanically competent horizons. Pressuremeter testing provides direct estimates of soil stiffness and limit pressure for foundation design, while ERT allows the detection of conductive clay-rich zones and resistive carbonate or rock bodies in complex geological environments. Despite these advances, most existing studies focus on relatively homogeneous soil conditions or rely on a limited combination of methods. Consequently, highly heterogeneous clay–carbonate systems, particularly in slope-controlled and seismically active environments, remain insufficiently investigated.

In addition, northern Algeria is located within an active tectonic context associated with the convergence between the African and Eurasian plates. This tectonic setting generates moderate to high seismicity, according to the Algerian seismic design code (RPA 99/2003), which can significantly influence geotechnical behavior. Recent studies have shown that complex fault systems and distributed deformation patterns can enhance high-frequency ground motion, thereby increasing the potential for slope failure and ground deformation even during moderate magnitude earthquakes. The 2020 Mila seismic sequence provides a clear example of such conditions, where ground shaking triggered landslides and structural damage in already vulnerable areas.

Despite the increasing number of studies, important gaps remain in the understanding of subsurface conditions and their engineering implications in the Mila Basin. In particular, the integration of geotechnical, geophysical, and hydrogeological data remains limited, especially for capturing lateral variability, in situ mechanical behavior, and transient hydrological effects. Furthermore, the combined influence of clay mineralogy, gypsum content, surface-water processes, and seismic loading on foundation performance has not been systematically quantified in this region.

In this context, the present study aims to provide a comprehensive and integrated characterization of the Sanaoua site by combining geotechnical, geophysical, and hydrogeological investigations within a unified engineering framework. The objectives are to: (i) characterize near-surface stratigraphy and its lateral variability; (ii) quantify in situ stiffness and strength parameters using pressuremeter and dynamic penetration tests; (iii) assess groundwater conditions during a representative wet-season monitoring period; and (iv) derive practical engineering interpretations for foundation design and urban development. Beyond presenting experimental results, the study proposes a simplified and field-applicable geotechnical model that can support land-use planning, foundation design, and risk mitigation strategies under both climatic and seismic constraints.

## Material and methods

### Site setting, morphology, and geomorphological constraints

**Fig. 1:**
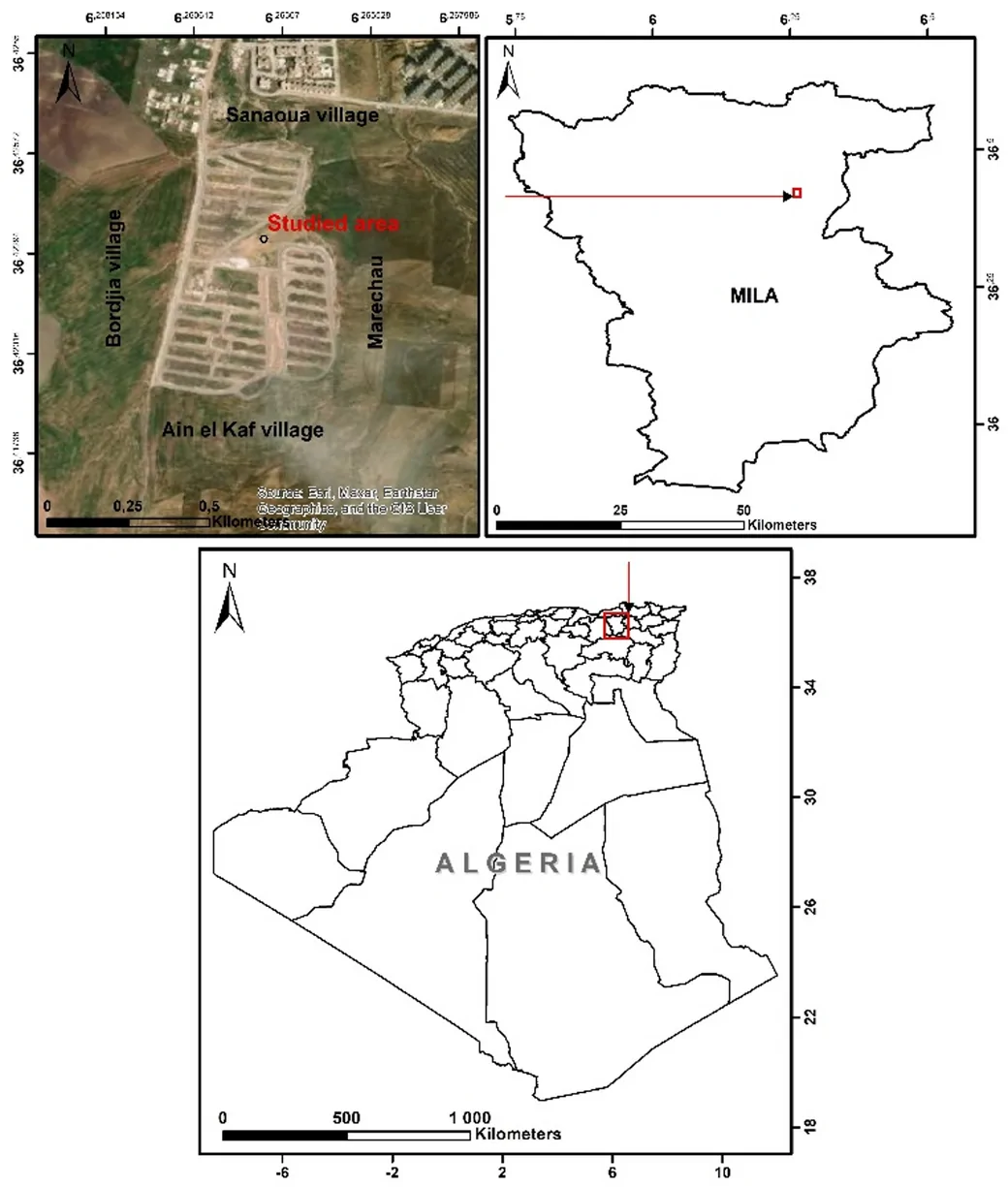
Location map of studied site. Base map was generated using Sentinel-2 imagery (Copernicus Programme, ESA) and Landsat data (USGS). Image processing and map production were carried out using ArcGIS (version 10.0.0.2414, https://www.esri.com).

The Sanaoua site (≈ 40 ha) is located on the western foothills of Mount Marechau (1256 m), approximately 5 km south of Mila City. It is bounded to the north by the village of Sanaoua, to the east by the road leading to Marechau, to the south by Ain El Kaf village, and to the west by Bordjia village (Fig. 1). The area is characterized by a concave hillslope morphology dissected by seasonal drainage channels (thalwegs), with an overall slope inclination toward the north. Field observations reveal active surface-water erosion and multiple landslide features aligned with the downslope direction, indicating that runoff concentration and slope-controlled mass movements constitute major constraints on urban development^7,12^. These geomorphological characteristics are consistent with ongoing incision processes and sediment transfer toward Oued Mila and the Beni Haroun Dam^13^.

### Geo-environmental conditions

Mila City and its surrounding expansion areas are located at the foothills of Mount Marechau, within a broad intramontane depression. The landscape is characterized by irregular morphology, including hummocks, bulges, and ridges, with slope gradients generally ranging from 7 to 16% and locally exceeding 25% along a south–north orientation^14,15^. The area experiences a temperate and humid climate^16^ and is underlain predominantly by clayey to marly formations of Mio–Plio–Quaternary age^10^.

The region is transected by a major SE–NW-oriented fault system linking the eastern Sidi Khelifa–Terai Beinane sector along the Ain Tine–Mila (El Kherba)–Zeghaïa alignment^13^. In addition, the area is characterized by moderate to high seismicity, according to the Algerian seismic design code (RPA 99/2003), which classifies the Mila region within seismic zone IIa–IIb. This corresponds to peak ground acceleration (PGA) values of approximately 0.15–0.25 g. This classification is consistent with regional seismic hazard assessments and reflects the tectonic activity associated with the convergence between the African and Eurasian plates^17,18^. In 2020, it was affected by several earthquakes, including an event of magnitude M = 4.6 on July 17th, followed by events of magnitudes M = 4.8 and M = 4.4 on August 7th. These latter events triggered a major landslide in the western extension of Mila City (El Kherba site), resulting in building collapses, severe structural damage, displacement of residents, and disruption of gas and water supply networks^4,13,19,20^. In addition to controlling regional tectonic deformation, fault-network geometry may also influence rupture behavior and the spectral content of seismic shaking. Recent studies have shown that structurally complex fault systems tend to generate stronger high-frequency ground motions than expected and may be associated with higher corner frequencies and stress drops, with potentially important implications for the seismic response of clay-rich slopes and foundation soils^21,22^.

The expected geotechnical consequences of earthquakes in the region—particularly slope instability, rockfall, and soil fracturing—are controlled not only by the peak ground motions but also by the frequency content and duration of seismic shaking. Recent seismic source studies indicate that fault network geometry influences the earthquake rupture process, such that geometrical complexity (e.g., bends, branches, step‑overs, and interacting fault strands) enhances high‑frequency seismic radiation and stress drop variability relative to simpler fault configurations. These high‑frequency components strongly contribute to the dynamic stresses in shallow soils and slopes, increasing the propensity for brittle near‑surface failures.

Furthermore, regional seismicity catalogs for the Western Mediterranean and North African tectonic domain reveal that seismogenic deformation is accommodated across networks of closely spaced, interacting faults rather than isolated major structures. This distributed seismicity, coupled with geometrical complexity, affects the spectral content of ground motion: fault systems with greater geometrical irregularity tend to produce rupture spectra with enhanced high‑frequency energy, which is more efficient at exciting short‑wavelength ground responses that are critical for slope destabilization and damage to built features.

Collectively, these processes imply that slope damage and related geotechnical impacts following events like the 2020 Mila earthquakes are not solely a function of magnitude and distance, but are also modulated by the geometry and complexity of the underlying fault network that ruptured, shaping the high‑frequency ground motion that drives shallow failure mechanisms.

### Method

**Fig. 2:**
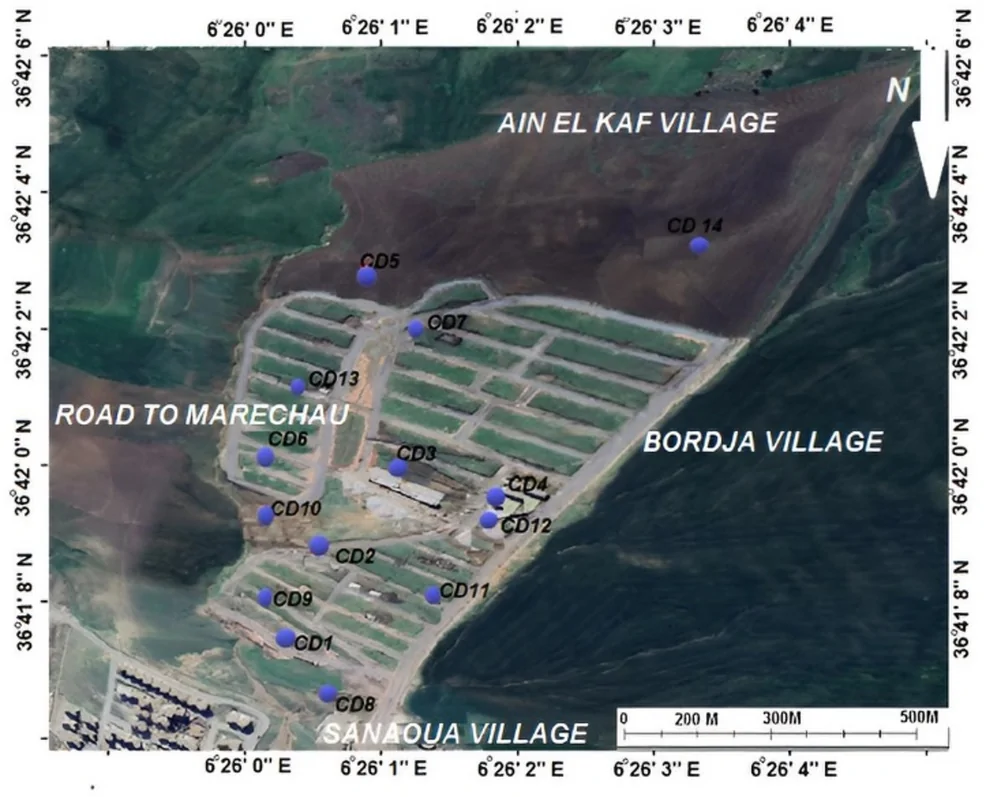
Location map of core drilling points (CD) in the Sanaoua area (Mila Basin, northeastern Algeria). Base map was generated using Sentinel-2 imagery (Copernicus Programme, ESA) and Landsat data (USGS). Image processing and map production were carried out using ArcGIS (version 10.0.0.2414, https://www.esri.com).

**Fig. 3:**
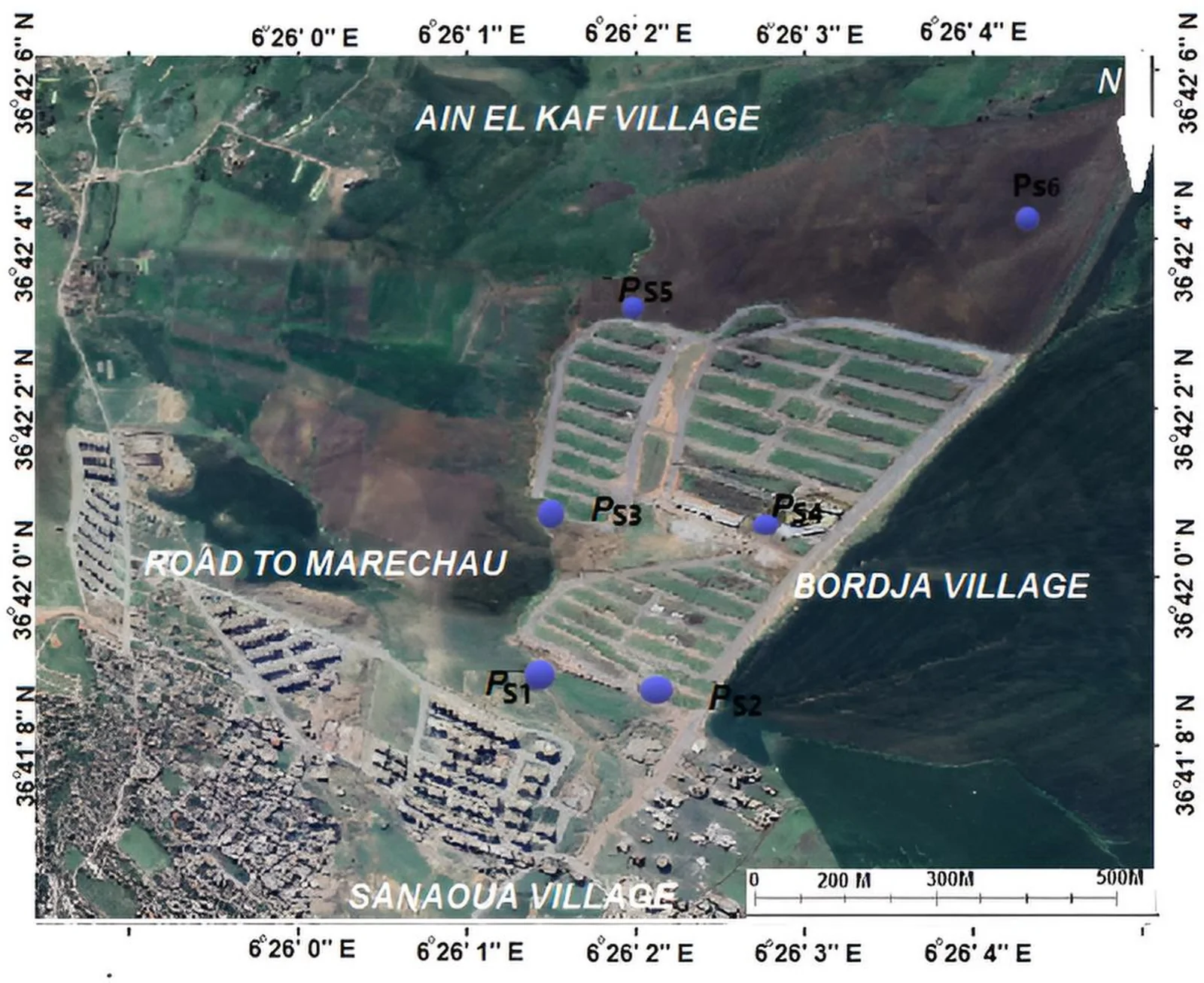
Location map of pressuremeter testing (Ps) in Sanaoua area. Base map was generated using Sentinel-2 imagery (Copernicus Programme, ESA) and Landsat data (USGS). Image processing and map production were carried out using ArcGIS (version 10.0.0.2414, https://www.esri.com).

**Fig. 4:**
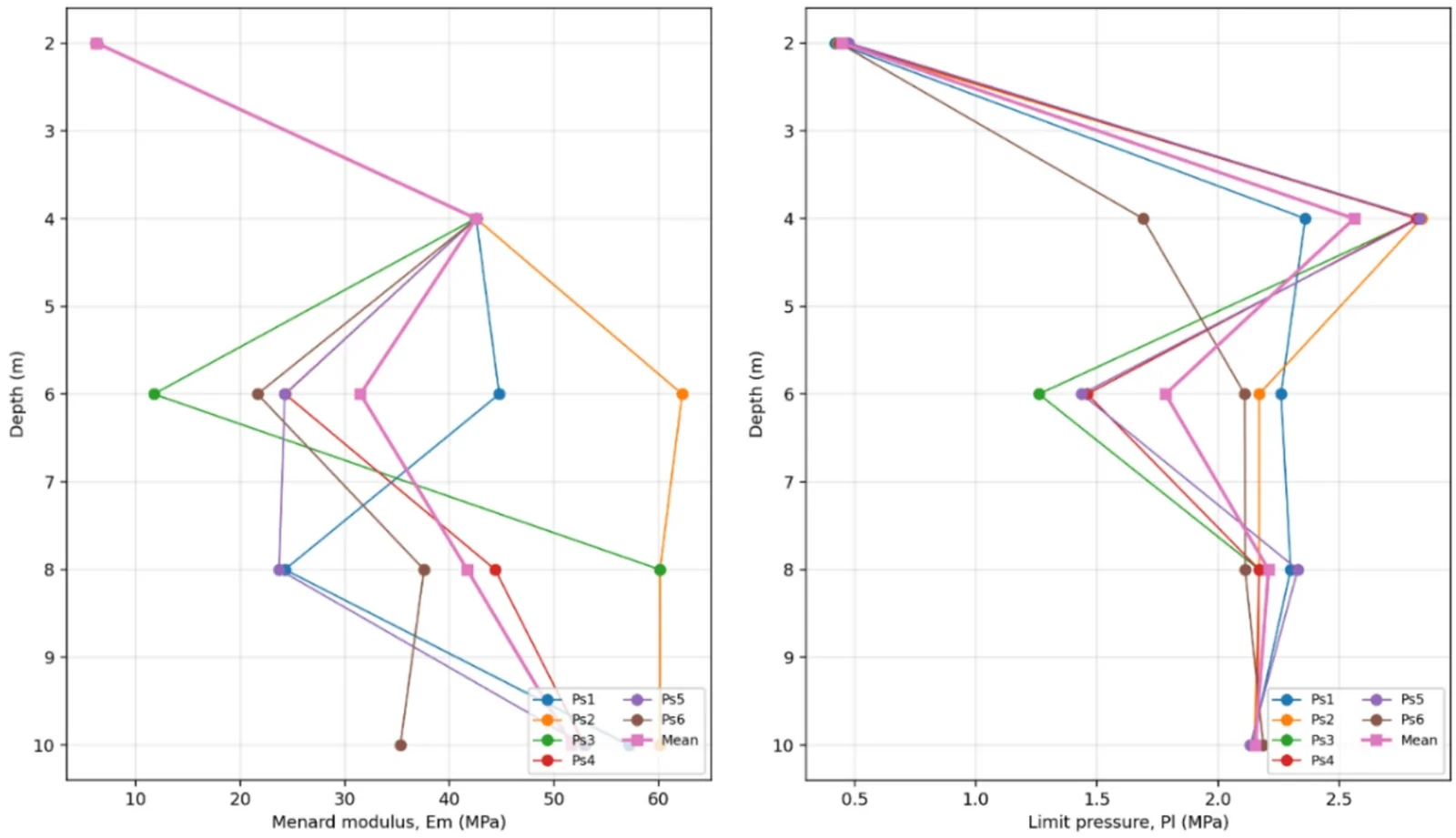
Variation of limit pressure (P_*l*_) and Menard modulus (E_m_) with depth.

**Fig. 5:**
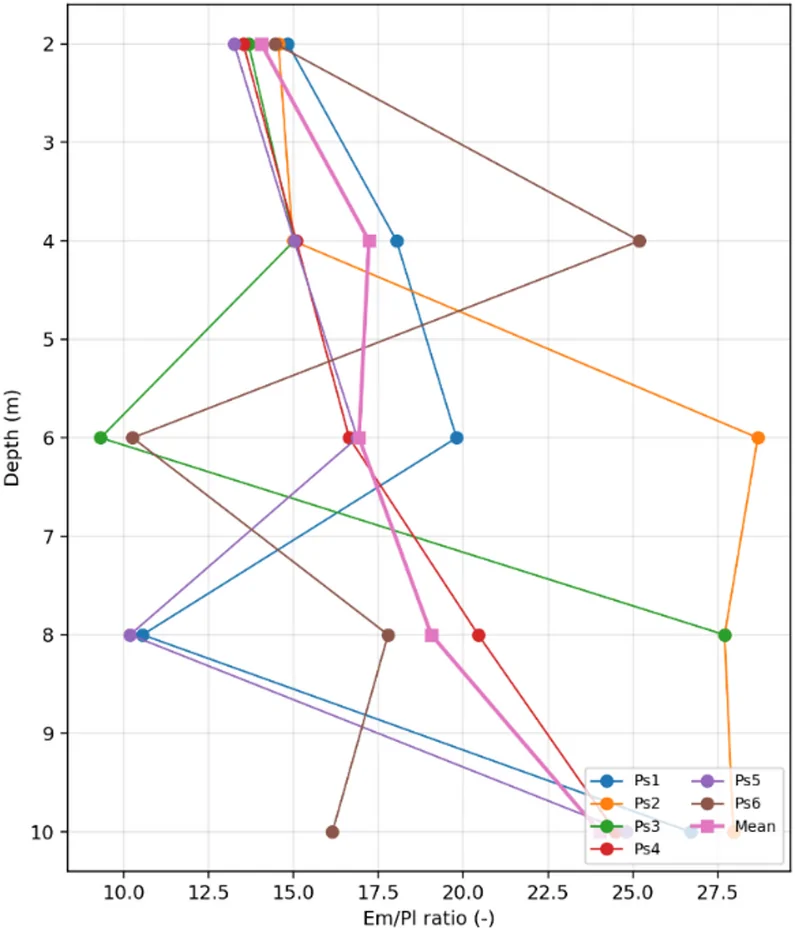
Variation of E_m_/P_*l*_ ratio with depth.

**Fig. 6:**
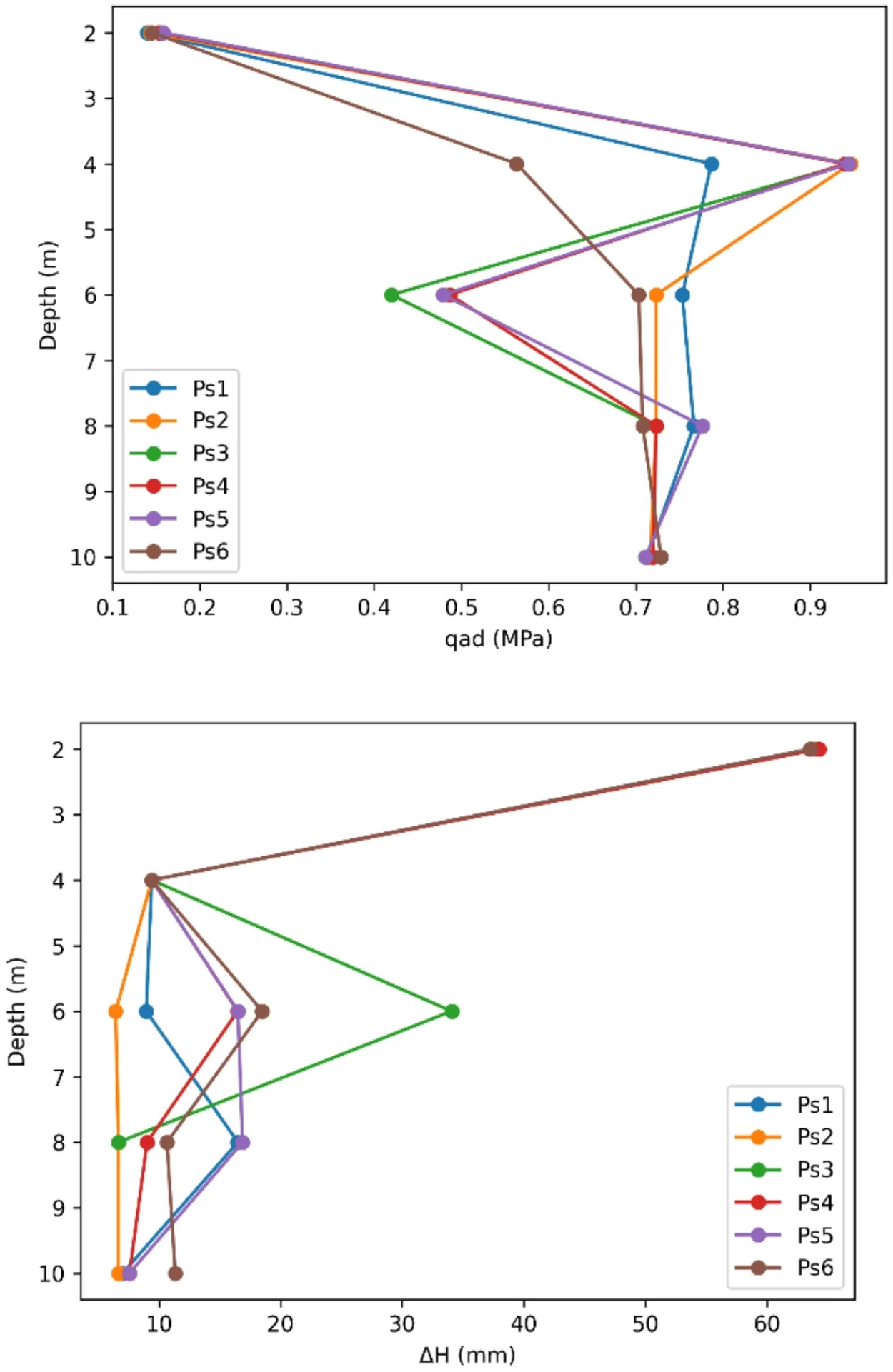
Variation of admissible bearing-capacity (q_ad_) and settlements (Δ*H*) with depth. This Figure clearly shows that bearing capacity increases significantly with depth, while settlement decreases. This trend confirms that shallow layers are unsuitable for foundation support, whereas deeper horizons provide more stable and reliable conditions.

This study adopts an integrated geotechnical investigation strategy to develop a practical engineering model based on the Mohr–Coulomb constitutive framework, adapted to the heterogeneous deposits of the northern expansion zone of Mila City, with particular emphasis on the Sanaoua site. The investigation program comprised the following components :

- Fifteen (15) core drillings (CD) were performed to depths of up to 10 m using an ABYSS 50 drilling rig equipped with the Geo Tool system (Fig. 2). Soil and rock descriptions followed^23–25^, standards, allowing standardized identification of clay layers, definition of stratigraphy, recognition of soil–rock transitions, and collection of representative samples for laboratory testing. Borehole logs constituted the primary geological reference for interpreting subsurface heterogeneity and calibrating in situ and geophysical data.
- Six (6) Ménard pressuremeter tests (Ps) were conducted in auger-drilled boreholes in accordance with^26–28^ (Fig. 3). Pressure–volume curves were corrected for membrane resistance, pressure losses, and creep effects, and were used to determine the Ménard modulus (Eₘ), limit pressure (P_*l*_), and the E_m_/P_*l*_ ratio. Depth-dependent profiles of these parameters were generated by linear interpolation and subsequently applied in bearing-capacity and settlement analyses.
- Twenty-five (25) dynamic penetration tests (P) were carried out in accordance with^29,30^ (Fig. 4). The number of blows required to achieve 20 cm penetration was recorded and used to calculate minimum and maximum peak resistances (Pr(min) and Pr(max)). Competent layers and bedrock (BR) depths were identified based on refusal criteria defined in^25^ (penetration < 20 cm after 50 blows), enabling detailed mapping of penetration resistance, bearing-capacity variability, and depth to mechanically competent horizons.
- Ten (10) Electrical resistivity tomography (ERT) profiles were acquired using a Wenner–Schlumberger array along linear survey lines to obtain two-dimensional resistivity sections (Fig. 5). Each profile consisted of 24 electrodes with 3 m spacing. As apparent resistivity values cannot be directly interpreted in heterogeneous ground, the data were processed using inversion techniques implemented in Res2DInv software to derive true subsurface resistivity distributions.
- Ten (10) piezometers (Pz) were installed across the site to assess groundwater presence, depth, and temporal variations (Fig. 6). Water levels were monitored after stabilization and throughout the observation period, providing essential information for defining hydrogeological conditions, estimating effective stresses, and evaluating groundwater-related constraints on urban development.
- Laboratory analyses were conducted in accordance with^31–37^ standards to determine the physico-mechanical properties of the soils. The derived parameters were used for foundation design and settlement calculations based on classical geotechnical theory (Terzaghi) and interpreted with reference to^25^, Eurocode 7^38–40^.
- Although clay mineralogy plays a key role in controlling the mechanical and hydro-mechanical behavior of fine-grained soils, no additional X-ray diffraction (XRD) analyses were performed in the present study. This choice is justified by the availability of detailed mineralogical investigations previously conducted on the same geological formations in the Mila Basin^11,14^. These studies demonstrated that the clayey deposits are predominantly composed of expansive minerals, including smectite and illite, with variable gypsum content.

Given the consistency of lithological conditions across the study area, these existing mineralogical data are considered representative of the investigated site. Therefore, the present work focuses on in situ geotechnical characterization and geophysical investigation to assess spatial variability, mechanical behavior, and engineering implications, rather than duplicating mineralogical analyses already well established in the literature.

- All investigation results were compiled into a unified database and jointly interpreted. Core drilling data defined the main geotechnical units, while dynamic penetration results were compared with Eₘ and P_*l*_ values. Owing to weak correlations, pressuremeter and laboratory data were prioritized for design parameters. ERT profiles were cross-validated with borehole data to confirm layer continuity and thickness, and piezometric measurements constrained groundwater conditions and effective stress assumptions. On this basis, a conceptual geotechnical model was developed and implemented as a layered Mohr–Coulomb model incorporating unit weight (γ), cohesion (c′), friction angle (φ′), stiffness (E), Poisson’s ratio (ν), and groundwater conditions for numerical evaluation of deformation and bearing capacity in support of safe urban development at the Sanaoua site.
- Results were synthesized in tables, graphs, and spatially interpolated maps. Statistical analyses and graphical outputs were produced using Microsoft Excel and R software, while spatial interpolation and visualization of key geotechnical parameters were carried out using QGIS version 3.16 (https://qgis.org) and ArcGIS version 10.0.0.2414 (https://www.esri.com) to quantify and illustrate spatial variability across the study area

### Site access and permissions

All field investigations conducted in the Sanaoua sector (Mila basin, northeastern Algeria), including core drilling, pressuremeter testing, dynamic penetration tests, electrical resistivity tomography surveys, and piezometer installations, were carried out with the appropriate authorizations from the competent local administrative and land management authorities.

The investigated area is designated for planned urban expansion and falls under public jurisdiction. All investigations were performed in accordance with applicable national regulations governing geotechnical and geophysical site investigations, environmental protection, and public safety standards.

## Results and descussions

### Stratigraphy and near-surface variability from core drilling (CD)

The results of the geotechnical investigation conducted in the southern part of Mila City, based on core drilling data (Fig. 2), are summarized in Table 1. Figure 2 illustrating the spatial distribution of boreholes used to characterize subsurface stratigraphy and geotechnical variability across the study site. Table 1 shows that the subsurface profile consists mainly of a thin topsoil layer overlying thick gray clay deposits containing gypsum fragments and limestone blocks. This indicates a predominantly cohesive and heterogeneous formation, which is likely to exhibit variable mechanical behavior and significant.

**Table 1:** Description of vertical cross sections from some core drillings.

Lithology		
CD	Topsoil	Gray clays containing gypsum fragments and lacustrine limestone blocks
8	1–2 m	2–10 m
2, 3, and 7	0–2 m	2–10 m

Core drilling results reveal a two-layer stratigraphic sequence within the 0–10 m depth interval. A surficial topsoil–colluvial layer occurs at the surface with variable thickness, ranging from approximately 0.5–1 m in the eastern sector, to about 1.9–3 m in the central area, and 2–3 m in the western part of the site. Along the slope direction, this layer thins from approximately 3 m in the south to around 2 m in the central zone and about 1 m in the northern sector, reflecting significant erosion and/or reduced sediment accumulation downslope (Table 1). This spatial distribution (Fig. 2) supports the interpretation of a concave hillslope compartment bounded by thalwegs, where seasonal runoff erodes exposed areas and redistributes sediments.

The surficial deposits overlie gray clay formations containing gypsum fragments and blocks of lacustrine limestone. Within the investigated depth, the clay unit displays thickness variations consistent with progressive northward thickening, reaching approximately 7 m in the southern sector, 8 m in the central area, and up to 9 m in the northern part of the site (Table 1). This geometry indicates that compressible fine-grained materials dominate most foundation levels. From an engineering perspective, the combination of variable topsoil thickness, thick clayey deposits, and slope-controlled runoff concentration provides favorable conditions for gully development, shallow sliding along drainage lines, and differential settlement across cut-and-fill platforms. This lithological characterization is consistent with observations reported by Khellaf et al.^5^ and Bourenane and Mezouar^8^, who noted that these materials frequently become plastic and weathered, rendering them highly sensitive to changes in water content and prone to differential settlement.

The stratigraphic sequence identified in the 0–10 m depth interval reflects significant spatial variability in surficial deposits and deeper clayey formations, which has important geomorphological and geotechnical implications for the study area. The observed variability in topsoil–colluvial thickness, with greater accumulation in the western sector and thinning upslope toward the north, is consistent with patterns of soil redistribution in concave hillslope systems reported elsewhere^41,42^. Concave slope compartments are recognized as loci of sediment deposition, whereas steeper upslope segments are dominated by erosional processes^43^. The progressive thinning toward the northern sector suggests active downslope migration of fines under the influence of overland flow.

Runoff‑driven erosion and sediment transport have been shown to exert a first‑order control on soil thickness and landscape evolution in semi‑arid to Mediterranean environments analogous to the present site^44,45^. Moreover, fluctuations in seasonal rainfall intensity can exacerbate colluvial removal and gully expansion, especially where vegetation cover is sparse^46,47^. Consequently, the spatial pattern of topsoil colluvium observed here is not unique but aligns with established models of slope transport and erosion.

Beneath the surficial layer, the gray clay formations with gypsum fragments and lacustrine limestone blocks exhibit systematic northward thickening. Fine‑grained clay deposits are known for their high compressibility and sensitivity to changes in water content^48^. In Mediterranean and semi‑arid regions similar to northern Algeria, thick clay sequences frequently exhibit seasonal swelling and shrinkage, which leads to differential settlement and shallow heave^49,50^. The northward increase in clay thickness observed may thus reflect both depositional gradients in a former lacustrine setting^51^ and ongoing pedogenic processes that concentrate fine material downslope.

The engineering behavior of such materials has been extensively documented. For example, Mouazen^52^ notes that plastic clays with intermittent saturation cycles can suffer significant loss of strength, leading to slope instability. Similarly, Huang and Tsai^53^ report that compressible clays with interbedded evaporite fragments may experience localized shear deformation due to moisture fluctuations. These findings corroborate the potential for gully development and shallow sliding identified in the present study, particularly along drainage concentrators.

Furthermore, reported cases from Algeria and neighboring regions highlight analogous geotechnical challenges in thick colluvial‑clayey sequences. Bendifallah et al.^54^ observed that fine‑grained alluvial and colluvial deposits often exhibit low bearing capacity and high settlement potential, necessitating careful design of cut‑and‑fill platforms and foundation systems. The tendency for differential settlement detected here is consistent with these regional observations, reinforcing the need for site‑specific geotechnical characterization.

The lithological characteristics described align with work by Khellaf et al.^5^ and Bourenane and Mezouar^8^, who both reported the prevalence of plastic, weathered clays in stratigraphic sequences across similar topographic settings. Khellaf et al.^5^ emphasize the role of seasonal moisture dynamics in modulating clay plasticity and strength, while Bourenane and Mezouar^8^ demonstrate how fine‑grained materials contribute to differential settlements in engineered slopes and embankments. The present findings reinforce these conclusions and extend them by documenting systematic spatial variation in deposit thickness across the site.

The Sanaoua site is underlain by thick lagoonal–lacustrine clay deposits of medium plasticity (CL–CI), locally containing gypsum and limestone blocks rather than a continuous bedrock. These materials exhibit strong sensitivity to water-content variations, leading to plastic deformation and differential settlements^8^. From a geotechnical standpoint, the deposits behave as a deformable cohesive mass, for which shallow foundations are generally unfavorable. Consequently, deep foundation systems or soil-improvement techniques are recommended in heavily loaded or slope-sensitive areas, in accordance with Eurocode 7^38–40^.

This lithological assemblage is widespread throughout the Mila region, with thicknesses exceeding 20 m at the El Kherba site^11^ and more than 50 m in the eastern Marechau sector^5^. The presence of gypsum reflects deposition in a confined lagoonal environment^10^, locally enriched in manganese^14^. Furthermore, the occurrence of reworked Pliocene lacustrine limestone debris suggests that these deposits are of Quaternary age rather than Mio–Pliocene, as previously proposed. This interpretation is supported by the fact that Pliocene limestones cap the Neogene formations of the Mila–Constantine Basin and crop out at the summit of the Marechau Mountains^55^.

At Sanaoua, the combination of this stratigraphic architecture with stepped slopes, a humid climate characterized by intense rainfall, and a high-seismicity setting associated with a north–south-oriented fault system creates conditions highly susceptible to erosion, settlement, pore-pressure buildup, and slope instability. These factors necessitate careful geotechnical and hydrogeological evaluation prior to any engineering development.

### Soil characterisation based on pressuremeter testing (Ps)

#### In-situ deformability from Ps

The locations of the pressuremeter tests (Ps) are shown in Fig. 3. The measured soil deformation modulus (E_m_, Ménard modulus) and limit pressure (P_*l*_) values are presented as interpolated depth profiles in Figs. 4 and 5. Figure 3 illustrating the spatial distribution of in situ tests used to evaluate soil stiffness, limit pressure, and bearing-capacity characteristics across the site.

Figure 4 illustrates the increase of both E_m_ and Pl with depth, reflecting the progressive improvement in soil stiffness and strength. Figure 5 shows the variation of the E_m_/P_*l*_ ratio, highlighting the transition from soft near-surface materials to stiffer and more competent layers at greater depths.

Pressuremeter tests conducted at six locations (Ps1–Ps6) (Fig. 3) reveal a clear increase in stiffness and strength with depth across the site. The upper 2 m are characterized by very low Ménard modulus values (E_m_ ≈ 6.0–6.3 MPa) and low limit pressures (P_*l*_ ≈ 0.42–0.48 MPa). A pronounced improvement is observed from depths of approximately 4 m downward, where E_m_ generally increases to 4.0–6.2 MPa and P_*l*_ to 1.3–2.8 MPa (Fig. 4). At depths of 8–10 m, most profiles exhibit relatively high E_m_ values (≈3.5–6.0 MPa) (Fig. 4) and P_*l*_ values close to or exceeding 2 MPa, indicating stiff to very stiff materials with markedly improved mechanical competence. Localized decreases in E_m_ at intermediate depths, particularly at Ps3 and Ps6 around 6 m, reflect subsurface heterogeneity, likely related to lithological variations and changes in moisture conditions within the marly clay sequence.

Spatially (Fig. 3), the pressuremeter results display a broadly consistent stratigraphic pattern across the site, with lateral variability primarily controlled by topography and elevation. Lower-elevation locations (Ps1 and Ps2) exhibit a more rapid and continuous increase in stiffness with depth, whereas higher-elevation points located on steeper slopes (Ps5 and Ps6) show more irregular stiffness profiles. These irregularities likely reflect enhanced weathering effects and variations in groundwater influence. The vertical trends of E_m_ and P_*l*_, interpreted in accordance with^25–28^, are consistent with stiffness–pressure relationships reported in the literature. Comparable responses in stiff geomaterials have been documented by Rebiai and Guettala^56^, while depth-related increases in stiffness have also been observed in intercalated dispersible cherts^57^. According to the classification criteria of^26–28^, these trends indicate a transition from loose or soft materials near the surface to dense granular deposits or stiff to very stiff cohesive formations at greater depths. This interpretation is further supported by dynamic penetration test results, which show increasing penetration resistance (Pr) and bedrock depths of approximately 1.8 m and 9.6 m. These values are characteristic of stiff to very stiff soils or weakly weathered rock and are consistent with the lithological model of clay deposits containing limestone blocks.

Near the surface (0–2 m), low E_m_ and P_*l*_ values are associated with relatively high E_m_/P*l* ratios, generally ranging from approximately 13–15 (Fig. 5). These values are typical of soft to moderately stiff cohesive soils exhibiting a pronounced deformational response. At depths between 4 and 6 m, both E_m_ and P_*l*_ increase markedly, while E_m_/P_*l*_ ratios commonly range from about 14–20 (Fig. 5), indicating a transition to stiffer cohesive materials in which stiffness and strength increase in a balanced manner. These observations are consistent with the behavioural trends reported by Lopes Dos Santos^58^, who documented proportional depth-related increases in both stiffness and limit pressure in soils tested using the Ménard pressuremeter, as well as with the findings of Rebiai and Guettala^56^ for stiff geomaterials. They are also in agreement with the broader interpretations proposed by Öge et al.^59^, who emphasized that weak, moisture-sensitive soils may display poor shallow mechanical performance and require careful depth-dependent interpretation, and by Lachérade et al.^60^, who showed that pressuremeter responses in heterogeneous formations must be interpreted with explicit consideration of spatial variability. In addition, the work of Nandi et al.^61^ supports the engineering relevance of Eₘ and Pl as robust in situ descriptors of deformability and strength, further confirming the value of pressuremeter data for identifying depth-related improvements in ground competence. Together, these studies support the interpretation of a progressive transition from medium-density granular or clayey layers near the surface to denser sands or stiff clay formations at greater depths, in accordance with the classification criteria of^28^. At greater depths (8–10 m), several profiles, particularly Ps1, Ps2, and Ps4, exhibit Eₘ/P_*l*_ ratios exceeding 20. According to^26–28^, such values are characteristic of stiff to very stiff cohesive soils or dense granular formations with reduced compressibility and improved stress–strain behaviour. Localized decreases in Eₘ/P_*l*_ ratios at certain depths and locations, notably at Ps3, Ps5, and Ps6, highlight the lateral and vertical heterogeneity of the marly clay formation containing limestone blocks. These variations likely reflect differences in block content, weathering intensity, and moisture conditions, and are consistent with the type of sector-based heterogeneity emphasized by Lachérade et al.^60^. Overall, the trends in E_m_/P_*l*_ ratios corroborate interpretations derived from E_m_ and P_*l*_ values considered individually, confirming a stratified yet heterogeneous deposit. Shallow layers are characterized by unfavourable deformability, whereas deeper horizons provide mechanically competent conditions suitable for foundation support, in accordance with^25^, Eurocode 7^38,39^.

These variations likely reflect differences in block content, weathering intensity, and moisture conditions, and are consistent with the type of sector-based heterogeneity emphasized by Lachérade et al.^60^. Overall, the trends in E_m_/P_*l*_ ratios corroborate interpretations derived from E_m_ and P_*l*_ values considered individually, confirming a stratified yet heterogeneous deposit. Shallow layers are characterized by unfavourable deformability, whereas deeper horizons provide mechanically competent conditions suitable for foundation support, in accordance with^25^, Eurocode 7^38,39^.

#### Bearing capacity (q_ad_) and settlement (***ΔH***) assessment using E_m_ and P_***l***_

**Fig. 7:**
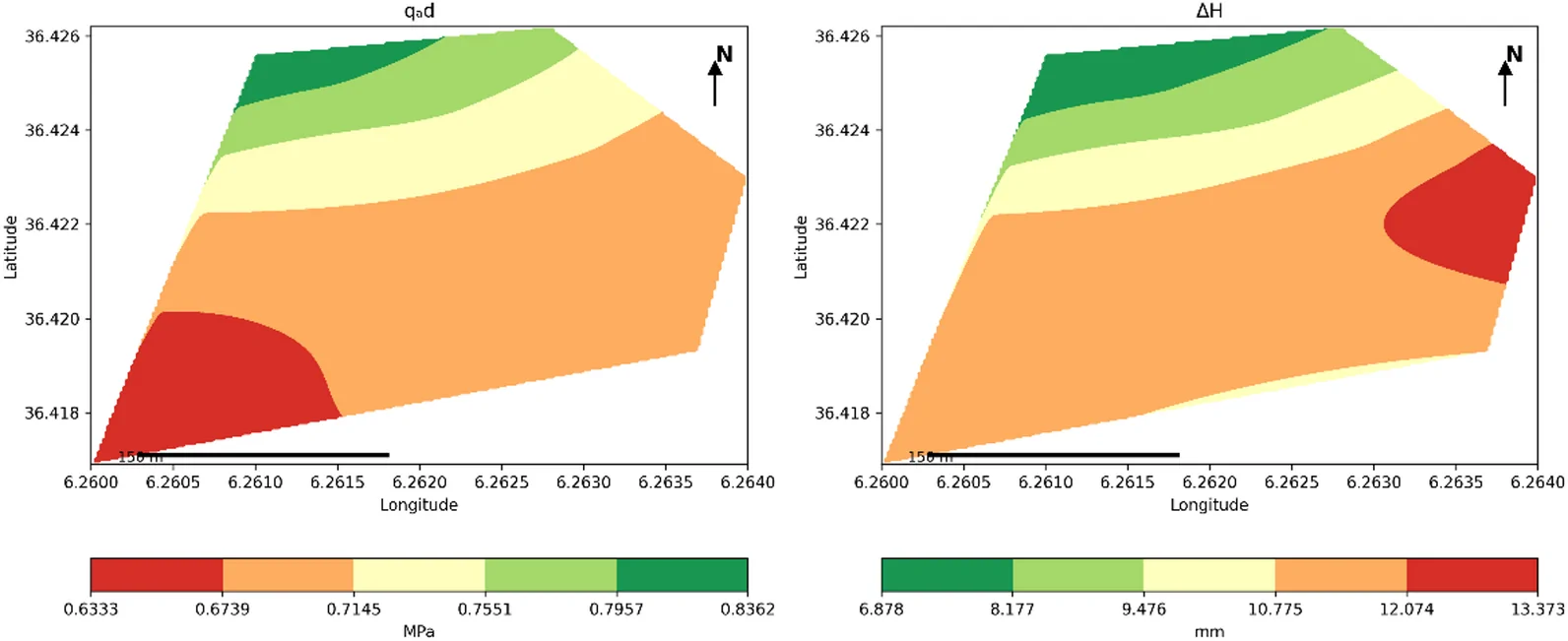
Spatial distribution maps of admissible bearing capacity (q_ad_, MPa) and estimated settlement (Δ*H*, mm) across the Sanaoua site (Mila Basin, northeastern Algeria), derived from pressuremeter test data. The results reveal marked spatial variability, with zones of low bearing capacity corresponding to higher settlement values, reflecting the heterogeneous nature of the clayey deposits and their implications for foundation design. Base map was generated using Sentinel-2 imagery (Copernicus Programme, ESA) and Landsat data (USGS). Image processing and map production were carried out using QGIS (version 3.16 https://qgis.org).

For the assessment of admissible bearing capacity (q_ad_), three standard Ménard formulations (q_ad_ = P_*l*_/3, q_ad_ = P_*l*_/4, and q_ad_ = P_*l*_/5) were applied, corresponding to optimized, conventional, and conservative design assumptions, respectively. This approach bounded evaluation of q_ad_ while accounting for depth-dependent variations in soil deformability. For the settlement (Δ*H*) analysis, a foundation width (B) of 2 m was adopted, as it is representative of typical shallow foundations used in standard building construction and is therefore suitable for characterizing the general site response. A design net stress (q) of 0.2 MPa was selected to maintain consistency with the measured soil stiffness, and a shape factor (α) of 0.9 was applied. Settlement curves (Δ*H*) were then derived using the combined parameters q, B, and E_m_. The results obtained for both admissible bearing capacity and settlement were spatially interpolated (Fig. 7) and are presented as depth-dependent profiles in Fig. 6.

The pressuremeter-based evaluation reveals a clear depth-dependent improvement in both admissible bearing capacity (q_ad_) and settlement behaviour (Δ*H*). Admissible bearing capacity values calculated using the three standard Ménard formulations are low within the upper 2 m, where q_ad_ typically ranges from 0.13 to 0.16 MPa using the P_*l*_/3 expression and decreases to approximately 0.08–0.10 MPa under conservative assumptions (P_*l*_/5) (Fig. 6). These values indicate insufficient bearing capacity for shallow foundations in the near-surface clayey layers. At intermediate depths of 4–6 m, q_ad_ increases markedly, reaching approximately 0.60–0.85 MPa (P_*l*_/3), 0.45–0.65 MPa (P_*l*_/4), and 0.35–0.50 MPa (P_*l*_/5) (Fig. 6). This increase reflects the mobilization of stiffer horizons within the marly clay formation. At greater depths (8–10 m), q_ad_ values generally stabilize between 0.70 and 0.90 MPa (P_*l*_/3), while even conservative estimates remain above 0.45–0.55 MPa (P_*l*_/5) (Fig. 6). These results indicate the presence of mechanically competent strata with reliable bearing capacity. Accordingly, the deeper horizons represent the most competent and reliable bearing layers, whereas the shallow strata, characterized by significantly lower P_*l*_ values, exhibit inadequate bearing resistance and increased susceptibility to deformation. The admissible bearing capacities derived at depth for the present site fall within the same general range as those reported by Rebiai and Guettala^56^, who identified pressuremeter-based bearing capacities characteristic of weak rock foundations. Comparable values were also documented by Hany Mostafa^57^, who demonstrated that jointed chert formations commonly display similar pressuremeter-derived bearing-capacity ranges. This overall consistency suggests that the marly substratum at the Sanaoua site exhibits bearing behaviour comparable to that of weak rock materials.

The settlement (Δ*H*) analysis exhibits a complementary depth-dependent trend. For a representative foundation width of B = 2 m, a design net stress of q = 0.20 MPa, and a shape factor α = 0.9, calculated settlement values are high within the upper layers, reaching approximately 60–65 mm at a depth of 2 m. These values clearly exceed acceptable serviceability limits for conventional structures. At depths between 4 and 6 m, Δ*H* decreases substantially to values generally ranging from 8 to 20 mm, reflecting the pronounced increase in the Ménard modulus (E_m_). At depths greater than approximately 6–8 m, settlement values are further reduced and commonly fall below 10 mm (Fig. 6), indicating favourable deformation behaviour. Localized increases in Δ*H* at certain depths reflect the inherent heterogeneity of the clay deposits containing limestone blocks; however, the overall trend remains consistent across the site. The settlement behaviour observed at the Sanaoua site, particularly the limited immediate deformation within the stiff marly clay layers, is consistent with the trends reported by Lopes Dos Santos^58^, who emphasized that soils characterized by high stiffness parameters generally undergo only minor instantaneous settlements under applied loads. These observations are further supported by the findings of Rebiai and Guettala^56^, who documented restricted deformation in stiff geomaterials, as well as by the results of Hany Mostafa^57^, where jointed chert and weak-rock units exhibited similarly controlled settlement responses under comparable loading conditions. This convergence of results supports the classification of the deeper strata as low-compressibility, rock-like materials. Overall, the settlement analysis indicates that only the deeper layers possess sufficiently high stiffness (elevated E_m_) to effectively limit deformation under typical structural loads, whereas the upper layers remain excessively compressible and unsuitable for direct foundation support.

Interpreted in accordance with^25–27^, these results confirm the presence of a vertically stratified soil profile in which both stiffness and strength increase with depth. When assessed against the ultimate and serviceability limit state requirements of Eurocode 7^39^ and the design principles of^38^, the analyses indicate that shallow foundations placed within the upper 2–4 m are controlled by both low admissible bearing capacity (q_ad_) and excessive settlement (Δ*H*), regardless of the design assumption adopted. In contrast, foundation solutions extending to depths that mobilize higher P_*l*_ and E_m_ values, typically between 6 and 10 m, are capable of satisfying both ULS and SLS criteria. The combined application of the three Ménard bearing-capacity formulations thus provides a robust and code-compliant framework for managing design uncertainty and for identifying depth intervals at which foundation performance becomes both mechanically reliable and serviceable. For design applications, the Ménard modulus (E_m_) should be directly incorporated into serviceability limit state calculations. In addition, foundation design must account for site-specific constraints, including steep slopes, a humid climatic regime, and the high seismicity (according to the Algerian seismic design code (RPA 99/2003) of the region.

### Dynamic penetration response and implications for “competent horizon” depth

**Fig. 8:**
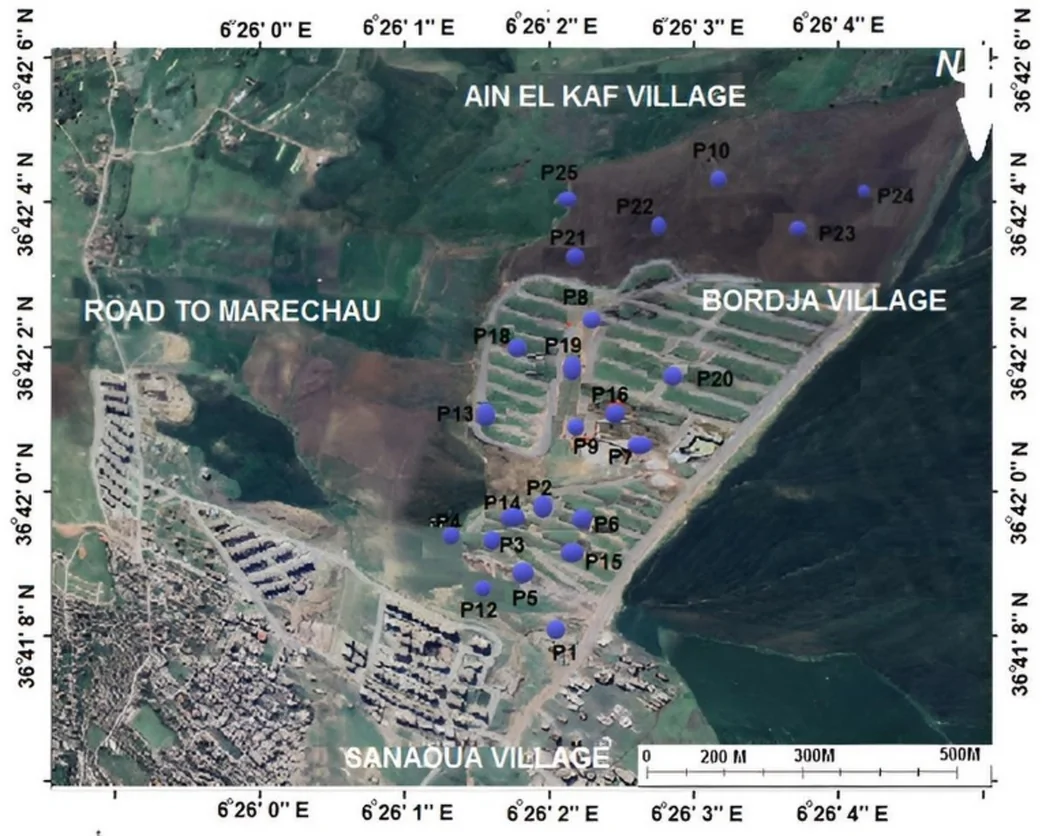
Location map of dynamic penetration testing (P) in Sanaoua area. Base map was generated using Sentinel-2 imagery (Copernicus Programme, ESA) and Landsat data (USGS). Image processing and map production were carried out using ArcGIS (version 10.0.0.2414, https://www.esri.com).

**Fig. 9:**
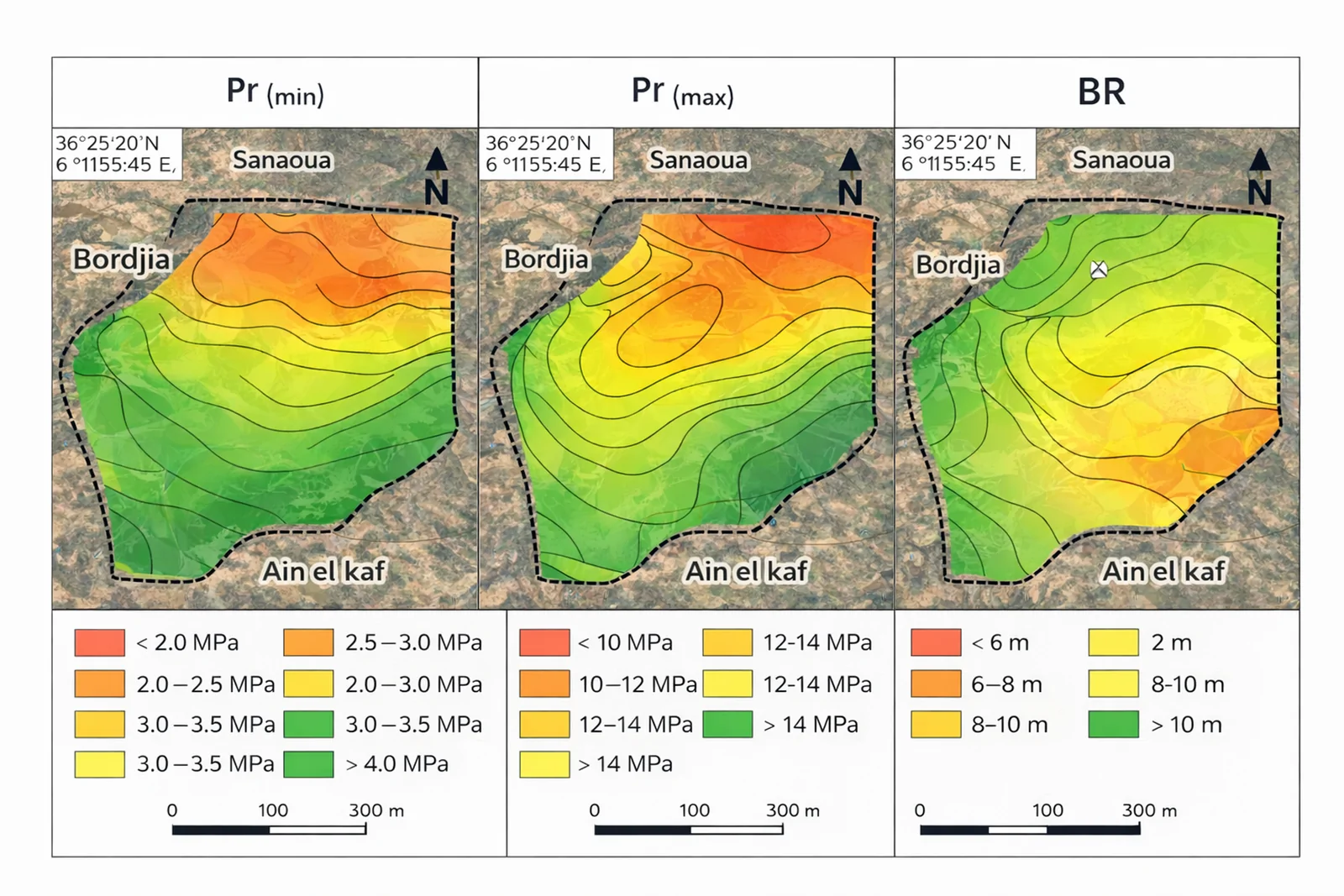
Spatial distribution of Pr(min), Pr(max), and BR results at the Sanaoua site. Image processing and map production were carried out using QGIS (version 3.16 https://qgis.org).

The locations of the dynamic penetration tests (P) are shown in Fig. 8. The obtained results are summarized and spatially interpolated in the maps presented in Fig. 9. Figure 8 illustrating the spatial distribution of in situ tests used to assess penetration resistance, soil stratification, and the depth to competent layers across the site. Figure 9 including minimum resistance (Pr(min)), maximum resistance (Pr(max)), and depth to bedrock (BR). The maps highlight significant lateral variability in soil resistance and bedrock depth, reflecting heterogeneous subsurface conditions across the study area.

The measured penetration resistance values (Pr(min) ≈ 1.7–6 MPa and Pr(max) ≈ 6.5–18 MPa) (Fig. 9) indicate that the Sanaoua site is predominantly composed of stiff soils and weathered to moderately competent rock. Most Pr(min) values fall within the range of 2–3 MPa, which is characteristic of medium to stiff clayey materials. Locally lower values occur, particularly in the southern sector (Figs. 8, 9), reflecting more compressible soils associated with enhanced weathering, fracturing, or higher moisture content, and therefore a greater settlement potential. The Pr(max) values frequently exceed 10 MPa and locally reach 15–18 MPa in the central and southern parts of the area (Figs. 8, 9), indicating the presence of competent subsurface horizons such as limestone blocks or partially cemented layers at depth. In contrast, more moderate Pr(max) values in the northern sector suggest thicker weathered profiles or the presence of structural discontinuities (Figs. 8, 9). Overall, the spatial distribution of these parameters (Fig. 9) highlights pronounced lateral variability in geotechnical conditions across the site. These results are consistent with international geotechnical standards and confirm a mechanically stratified subsurface, with strength and stiffness increasing with depth. According to^25,29,30^, the observed penetration resistance values reflect alternating layers of very soft to soft cohesive soils, moderately resistant materials, and locally very dense or blocky horizons. Local refusal or sharp increases in resistance after 100–150 blows per 20 cm^28^ indicate the presence of limestone blocks, consistent with lithological observations reported by Khellaf and Hamimed^11^, Khellaf^14^, and Khellaf et al.^5^. Although peak resistance values are lower than those reported for the Marechau sector^8,11^, they are comparable to results obtained in similar soil conditions^62^. Under^29,30^, the Sanaoua deposits can therefore be classified overall as compact to very compact ground, with enhanced bearing capacity related to partial cementation and the presence of limestone blocks.

From an engineering perspective, low Pr(min) values delineate very weak zones in which shallow foundations are susceptible to excessive settlement or punching-type failure. In contrast, high Pr(max) values and shallow bedrock depths indicate stiff to very stiff soils or weakly weathered rock capable of providing significantly improved foundation support. The pronounced lateral and vertical variability observed across the site implies rapid changes in bearing capacity and stiffness over short distances, thereby increasing the risk of differential settlement in uniformly founded structures. Accordingly, and in line with^39^(Eurocode 7)^38,40^,, areas characterized by low Pr(min) values should be avoided for heavily loaded structural elements or treated using appropriate ground-improvement techniques. Alternatively, such zones may be bridged by raft foundations or deep foundation systems. Furthermore, the spatial heterogeneity of Pr(max) values supports the implementation of geotechnical zoning based on dynamic penetration test results and the adoption of flexible foundation solutions, such as variable footing depths, combined footings, rafts, or piles, to accommodate local variations in ground conditions.

The depth to bedrock (BR) at the site ranges from approximately 4 m to more than 10 m, exhibiting pronounced lateral and vertical variability typical of hilly, slope-controlled terrains^38,39^. Spatially, bedrock depths generally exceed 9–10 m in the northern sector of the area, decrease to intermediate values of approximately 7–9 m in the central zone, and become relatively shallow (around 4–6 m) in the southern and south-western sectors (Fig. 9). This pattern is comparable to bedrock-depth distributions reported in nearby sectors of Mila^5,8,11,14^. Bedrock depth was identified where penetration resistance exceeded N 50 blows per 20 cm and penetration was less than 20 cm after 50 blows, in accordance with^25,29,30^. On this basis, three bedrock-depth classes can be distinguished. Shallow bedrock (≈ 4–6 m) is mainly associated with higher-elevation zones, reflecting erosional thinning of the weathered mantle. Deeper bedrock levels (≈ 8–10 m) occur in lower and intermediate slope positions, where colluvial accumulation and prolonged weathering have produced thicker weathered profiles. The northern and central sectors generally exhibit intermediate bedrock depths (≈ 6–9 m), whereas the southern and southeastern areas are characterized by thicker weathered zones. In accordance with^25,63^, this spatial distribution highlights the strong influence of topography and weathering processes and confirms the presence of a stratified subsurface composed of stiff soils to weathered rock overlying moderately competent bedrock. This interpretation is consistent with international standards for site characterization and provides a reliable basis for foundation design in the study area.

The combined spatial distribution of bedrock depth (BR), Pr(min), and Pr(max) values highlights pronounced lateral and vertical geotechnical variability across the Sanaoua site, with direct implications for engineering design. Shallow bedrock (≈ 4–6 m) in higher-elevation areas is generally associated with higher Pr(max) values, indicating stiff soils to weakly weathered rock that are suitable for shallow foundation systems. In contrast, deeper bedrock levels (≈ 8–10 m) in the lower and southern sectors correspond to lower Pr(min) and moderate Pr(max) values, reflecting the presence of thick weathered or clay-rich layers with reduced bearing capacity. This spatial heterogeneity implies rapid changes in stiffness and bearing capacity over short distances, thereby increasing the risk of differential settlement in uniformly founded structures. In accordance with^25,39,63^, such conditions necessitate the implementation of geotechnical zoning and the adoption of adaptable foundation solutions, including variable footing depths, raft foundations, or deep foundation systems in zones characterized by weaker ground conditions.

When correlated them with BR depth, Pr(min), and Pr(max) values, as well as ERT and core drilling (CD) data, the dynamic penetration test results confirm the presence of two main subsurface domains. The first is a conductive, clay-dominated horizon (engineering unit U2), characterized by low penetration resistance and reduced bearing capacity. The second is a resistive, carbonate-dominated domain (unit U3), which exhibits higher stiffness and more favourable geotechnical properties. The northern and central sectors of the site correspond to a U2–U3 transition zone, where limestone inclusions and compact marls locally provide conditions suitable for shallow to medium-depth foundations. In contrast, the southern and western parts of the site are dominated by thick clayey units associated with lower resistance values and greater bedrock depths, requiring foundation adaptation through ground-improvement measures or deep foundation systems such as micropiles. Interpreted admissible bearing capacities (q_ad_), derived from correlations with penetration resistance following Eurocode 7 and^25^, range from approximately 0.1–0.75 MPa, underscoring strong spatial variability in foundation suitability. The lateral alternation of clayey materials and carbonate blocks over short distances further indicates a significant risk of differential settlement, which must be carefully accounted for in foundation design and site development. Overall, these results support the implementation of geotechnical zoning across the site, distinguishing areas suitable for conventional construction from those requiring reinforced foundation solutions. They also emphasize the necessity of site-specific foundation design and stability assessment in this geomorphologically complex and erosion-prone environment.

### Geophysical prospecting and subsurface geometry

**Fig. 10:**
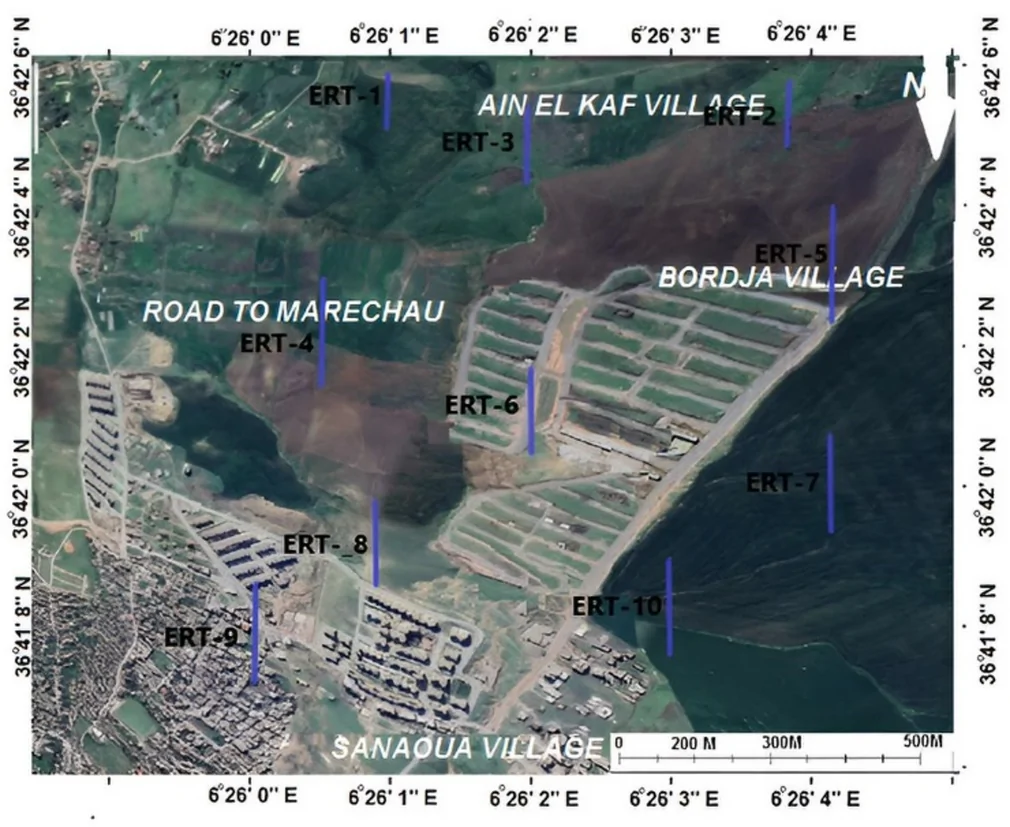
Location map of Electrical Resistivity Tomography (ERT) in Sanaoua area. Base map was generated using Sentinel-2 imagery (Copernicus Programme, ESA) and Landsat data (USGS). Image processing and map production were carried out using ArcGIS (version 10.0.0.2414, https://www.esri.com).

**Fig. 11:**
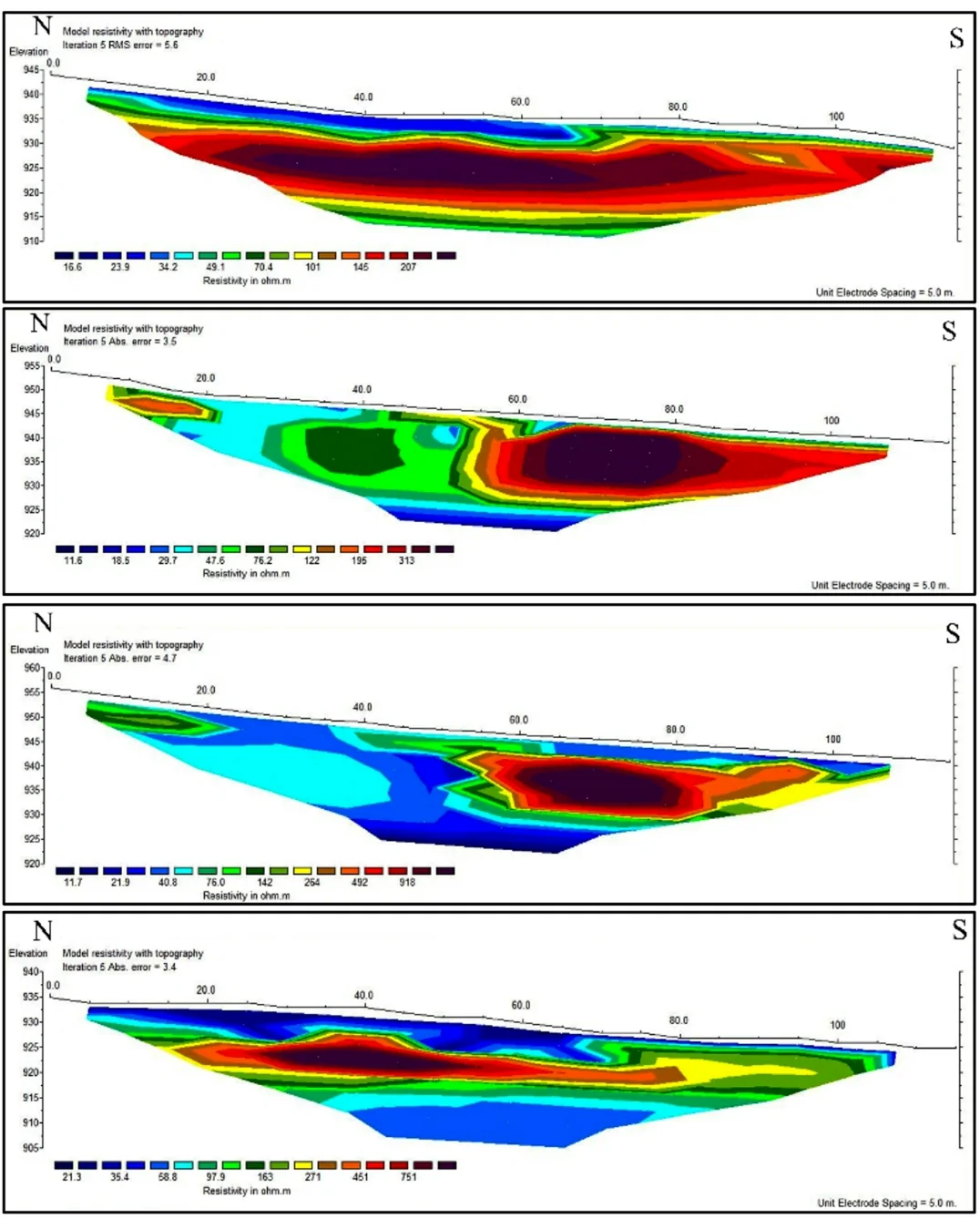
Some geo-electrical cross-sections for the Sanaoua site (PWD-Mila).

The locations of the electrical resistivity tomography (ERT) profiles within the study area are shown in Fig. 10. The results obtained from geophysical surveying and subsurface geometry interpretation are summarized in Fig. 11. Figure 10 showing the location and orientation of survey lines used to investigate subsurface resistivity and geological variability. Figure 11 illustrating the lateral and vertical variability of subsurface resistivity. Conductive zones (low resistivity) correspond to clay-dominated materials, while resistive anomalies indicate limestone blocks or competent carbonate horizons, highlighting the heterogeneous geotechnical conditions.

#### Resistivity classes linked to studied area

**Fig. 12:**
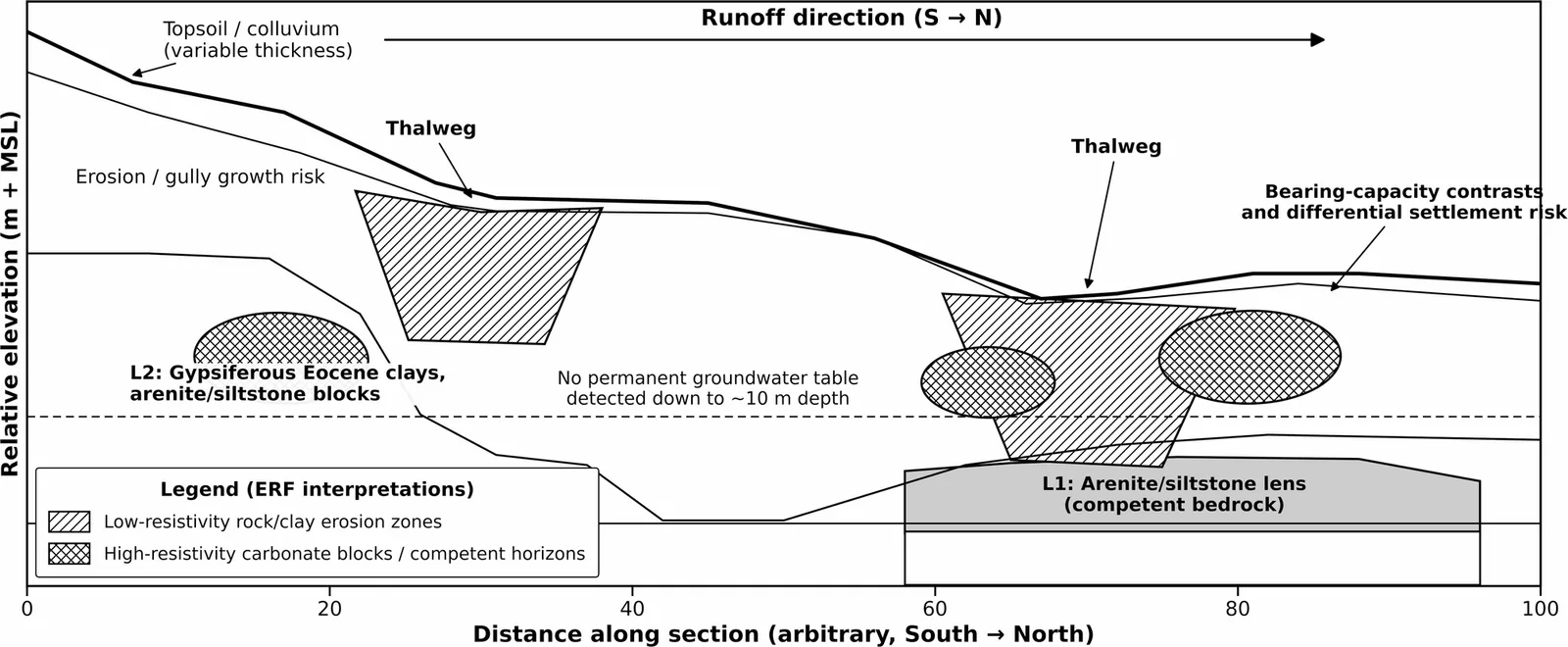
Conceptual 2D engineering geophysical geo-model diagram of the Sanaoua site.

The resistivity classes identified in the Marechau region are summarized in Table 2 and spatially interpolated to produce the geoelectrical model shown in Fig. 12. Table 2 presents the resistivity classes used to interpret the ERT data and links each resistivity interval to likely materials, engineering units, and geotechnical meaning. The table shows that low resistivity values are associated mainly with clay-rich, moisture-sensitive, and potentially unstable zones, whereas high resistivity values correspond to carbonate bodies or competent substrata that may provide better bearing conditions. Figure 12 showing the spatial organization of geotechnical units, including clay-rich conductive domains (U2) and carbonate-rich resistive horizons (U3), along with surface morphology and runoff direction. The model illustrates the influence of subsurface heterogeneity on erosion, slope instability, and foundation suitability across the site.

**Table 2:** Resistivity classes linked to Sanaoua engineering units.

Resistivity class (Ω.m)	Likely material (interpretation)	Engineering unit	Engineering meaning for Sanaoua
< 10	Wet/saturated clay; very conductive zones	U2 (locally)	Potential perched/transient water, softened clay pockets, higher instability sensitivity in storm periods^64^
10–40	Clay-rich soils/marls (often moisture-sensitive)	U2	Dominant gypsiferous clay mass; compressible; relevant for settlement and undrained stability^65^
40–150	Marl–marly limestone / clayey sand mixtures, drier horizons	Transition U1/U2 or U2/U3	Stiffer zones or mixed materials; may coincide with less plastic horizons or block-rich clay^65^
150–800	Limestone / hard carbonate (fractured to competent)	U3 (carbonate bodies)	Limestone blocks and/or competent carbonate horizon; may control shallow bearing and excavation difficulty^65^
800	Massive carbonate or dry, resistive rock; (pure gypsum can be high but needs caution)	U3 (if boreholes confirm)	Candidate “competent substratum”; confirm with drilling because gypsum can also show high values when pure/dry^65^

Accordingly, the ERT cross-sections from the Marechau region (Fig. 11) are expected to image: (i) a shallow, heterogeneous layer exhibiting moderate resistivity variability, corresponding to topsoil and colluvial deposits with variable moisture content; (ii) a thick, low-resistivity domain associated with high-plasticity clay formations (engineering unit U2), locally containing highly conductive zones related to transient water accumulation following rainfall events; and (iii) localized high-resistivity anomalies representing limestone blocks and/or a more competent carbonate horizon (unit U3), consistent with zones of penetration refusal and high peak resistance identified in dynamic penetration tests (Table 2; Fig. 12). The ERT results therefore corroborate the pronounced lateral heterogeneity observed in the penetration data and the variable depth of mechanically competent horizons. Moreover, they provide a spatial framework for delineating drainage corridors (thalwegs), guiding cut-and-fill planning, and anticipating excavation difficulty and bearing-capacity contrasts across the site.

#### Electrical resistivity tomography (ERT) results and subsurface engineering

The electrical resistivity tomography (ERT) results and their engineering interpretation are summarized in Table 3 which summarizes the main characteristics of the ERT profiles, including profile length, elevation range, dominant resistivity values, conductive zones, resistive anomalies, and engineering interpretation. This table highlights the strong lateral heterogeneity of the Sanaoua site, with alternating clay-dominated conductive sectors and carbonate-rich resistive sectors, confirming the variable geotechnical behavior observed across the area.

**Table:** 

Profile ID	Length (m)	Elevation investigated (m a.s.l.)	Vertical extent (m)	Dominant resistivity (Ω.m)	Low-ρ zones (< 40 Ω.m): elevation range(s)	High-ρ anomalies (150Ω.m): elevation range(s)	Interpretation (engineering units)
ERT-1	115	945 to 932.5	12.5	145 to 207	945 to 935 (surface); 915 to 913 (deep lens)	932.5 to 920	Dominant U3/transition U2–U3 (resistive carbonate/blocky zones), with a thin U1/U2 conductive cap at surface; local conductive pocket at depth (possible clay lens/perched moisture)
ERT-2	115	935 to 905	30.0	21.3 to 58.8	935 to 927.5 (surface); 917.5 to 905 (deep)	927.5 to 920 (localized, northern)	Predominantly U2 (clay), with a localized resistive body (U3) in the northern part; high potential for settlement/undrained response where low-ρ extends deep
ERT-3	115	932.5 to 905	27.5	149 to 204	932.5 to 925 (surface, northern part)	925 to 912.5	Mostly resistive domain (U3 / carbonate influence) beneath a conductive surface strip (U1/U2) in the north; consistent with blocky limestone / shallow competent horizon
ERT-4	115	956.5 to 922.5	34.0	11.7 to 40.8	956.5 to 922.5 (nearly whole section)	942.5 to 930 (localized, center)	Strongly U2 (thick conductive clay) across the full DOI; only a limited resistive core (U3) in the center. This is a key “soft-clay” sector for zoning
ERT-5	115	955 to 920	35.0	11.6 to 47.6 (north); 195 to 313 (south)	950 to 932.5 (surface, northern part)	942.5 to 930 (southern)	Clear north–south contrast: northern conductive U2 (clay) versus southern resistive U3 (carbonate/blocky or competent horizon)
ERT-6	117	932.5 to 902.5	30.0	116–152	922.5 to 920 (surface, northern part)	932.5 to 910	Dominantly resistive-to-intermediate material with strong resistive body (U3) extending through much of the section; only a thin conductive surface cap (U1/U2) locally
ERT-7	13.8	920 to 887.5	32.5	124 to 164	905 to 902.5 (surface, northern part)	920 to 887.5	Largely resistive domain (U3) across the full DOI with minor surface conductive cover (U1/U2). *Length “13.8 m” should be checked (often ~ 138 m in practice)
ERT-8	115	905 to 882.5	22.5	127 to 167	905 to 902.5 (surface, north + center)	905 to 885	Predominantly resistive U3 below a thin conductive cover (U1/U2) in north/center
ERT-9	92	900 to 877.5	22.5	126 to 173	895 to 877.5 (surface, north + center); 880 to 877.5 (deep, center)	900 to 880	Mostly resistive U3 at depth; conductive surface band in north/center and a deep conductive pocket centrally (clay lens / transient moisture zone)
ERT-10	115	990 to 955	35.0	12 to 20.7 (south); 61.3 to 536 (north)	970 to 955 (surface, center)	990 to 960	Very high heterogeneity: very resistive northern domain (U3, up to 536 Ω.m) contrasted with very conductive southern domain (U2). Resistive interval 990–960 indicates shallow competent/carbonate influence

The resistivity distribution at the Sanaoua site (Fig. 10) reveals two main geoelectrical domains that are consistent with the lithology defined from borehole data (Table 3): (i) a conductive domain (≈ 11–59 Ω.m) corresponding to clay-dominated materials and/or moisture-rich horizons, and (ii) a resistive domain (≈ 150–536 Ω.m) attributed to competent carbonate horizons and block-rich zones embedded within the clay formation.

Several ERT profiles are dominated by conductive behaviour. Profile ERT-4 provides the clearest example, with resistivity values of approximately 11.7–40.8 Ω.m extending through nearly the entire investigated thickness, indicating the presence of a thick clayey mass corresponding to engineering unit U2 in the central part of the site. Profile ERT-2 similarly reflects a predominantly clayey sector, with resistivity values ranging from 21.3 to 58.8 Ω.m, although a localized resistive anomaly in its northern portion suggests the presence of discontinuous competent carbonate features (unit U3). These conductivity-dominated profiles delineate priority zones for settlement-sensitive land-use planning, conservative short-term (undrained) stability assumptions, and stringent surface-water management measures. In contrast, other profiles are characterized by resistive dominance or pronounced resistive bodies. Profiles ERT-1 (145–207 Ω.m), ERT-3 (149–204 Ω.m), and ERT-6 to ERT-9 (typically 116–173 Ω.m, with extensive intervals exceeding 150 Ω.m) indicate the presence of extensive resistive formations corresponding to unit U3 or to U2–U3 transition zones, commonly overlain by thin conductive layers. This geoelectrical pattern is consistent with borehole observations of limestone blocks and with dynamic penetration test anomalies, including bedrock refusal and very high peak resistance values. These observations imply that the depth to a mechanically competent horizon is highly variable across the site and locally shallow. In profile ERT-9, the presence of a deep conductive pocket within an otherwise resistive section suggests a clay lens and/or a zone of transient moisture.

Pronounced lateral resistivity contrasts are also evident across the site. Profile ERT-5 displays a clear north–south partition, with a conductive northern sector (11.6–47.6 Ω.m) and a highly resistive southern sector (195–313 Ω.m), indicating rapid lithological and/or structural changes over short distances. Profile ERT-10 exhibits the strongest heterogeneity, characterized by a very conductive southern domain (12–20.7 Ω.m) and a highly resistive northern domain reaching values up to 536 Ω.m. This pattern is consistent with the juxtaposition of thick clayey units (U2) and a carbonate-controlled substratum or block-rich zones (U3). Overall, the ERT dataset confirms that the Sanaoua site does not constitute a laterally uniform clay platform. Instead, it comprises thick clay-dominated bodies locally interrupted by carbonate features that exert strong control on excavation conditions, bearing-capacity variability, and differential settlement. These interpretations are consistent with results reported by PWD-Mila (2015) and Khellaf et al.^5^ for the southeastern sector of the Marechau region accumulation. They are also in agreement with recent studies demonstrating that electrical resistivity tomography is an effective tool for identifying subsurface heterogeneity and geotechnical hazard conditions. In alluvial environments, ERT has been shown to delineate saturated and loose deposits associated with liquefaction potential and seismic amplification, particularly where shallow groundwater conditions prevail^66^. Similarly, combined geomorphological and geophysical analyses have demonstrated that strong resistivity contrasts may reflect structural controls such as faults or dissolution features, which govern the development of collapse-prone zones in heterogeneous terrains^67^. Furthermore, time-dependent and high-resolution resistivity investigations highlight the strong coupling between electrical properties, moisture variations, and mechanical behavior, showing that resistivity anomalies can indicate deformation, damage evolution, and fluid migration within geomaterials^68,69^. These findings reinforce the interpretation that the Sanaoua site is characterized by a highly heterogeneous subsurface, in which hydro-mechanical processes and lithological contrasts play a critical role in localized deformation processes and slope response.

The geophysical results correlate well with piezometric, geomorphological, and geotechnical observations across the study area and collectively indicate that slope instability and erosion processes are primarily governed by surface-water dynamics rather than by the presence of a persistent shallow groundwater. Electrical resistivity tomography (ERT) sections reveal a stratified subsurface composed of a resistive near-surface layer, an intermediate weathered horizon, and a deeper low-resistivity zone interpreted as clay-rich and locally water-saturated material. However, piezometric monitoring conducted at ten locations over a three-month autumn period did not identify a permanent groundwater within the investigated depth range, except for short-lived, film-type water stagnations. This apparent discrepancy can be explained by the low permeability and high plasticity of the Mio–Plio–Quaternary marly clays, as confirmed by laboratory test results indicating high liquid limits, high plasticity indices, and degrees of saturation frequently exceeding 90%. These properties favor the development of transient perched water conditions rather than sustained groundwater flow. Consequently, the basin-shaped conductive zones imaged by ERT are interpreted as zones of episodic saturation related to infiltration pulses, preferentially developing along thalweg infill deposits, permeability contrasts, and behind cut slopes, rather than representing continuous aquifer systems.

This interpretation is consistent with the concave hillslope morphology, the general northward-dipping topography, and the absence of springs or groundwater resurgences during the monitoring period. It is further supported by field evidence of active runoff erosion and downslope mass wasting directed toward Oued Mila and the Beni Haroun Dam. Pressuremeter results reinforce this conceptual framework by demonstrating weak, highly deformable near-surface layers overlying progressively stiffer marly clays and locally weathered bedrock at depth. Mechanical competence increases below approximately 4–6 m but remains laterally heterogeneous due to variable weathering intensity and moisture conditions. In this context, slope instability is most plausibly triggered by intense rainfall events that promote surface runoff concentration and transient pore-pressure increases within shallow clayey horizons, rather than by long-term groundwater pressures. Accordingly, mitigation and drainage strategies should prioritize the interception and controlled conveyance of surface runoff, limitation of infiltration into sensitive cut slopes, and prevention of localized ponding, while accounting for the potential role of short-lived perched water in triggering shallow instability and deformation processes.

### Hydrogeological behaveiour and role of transient water

**Fig. 13:**
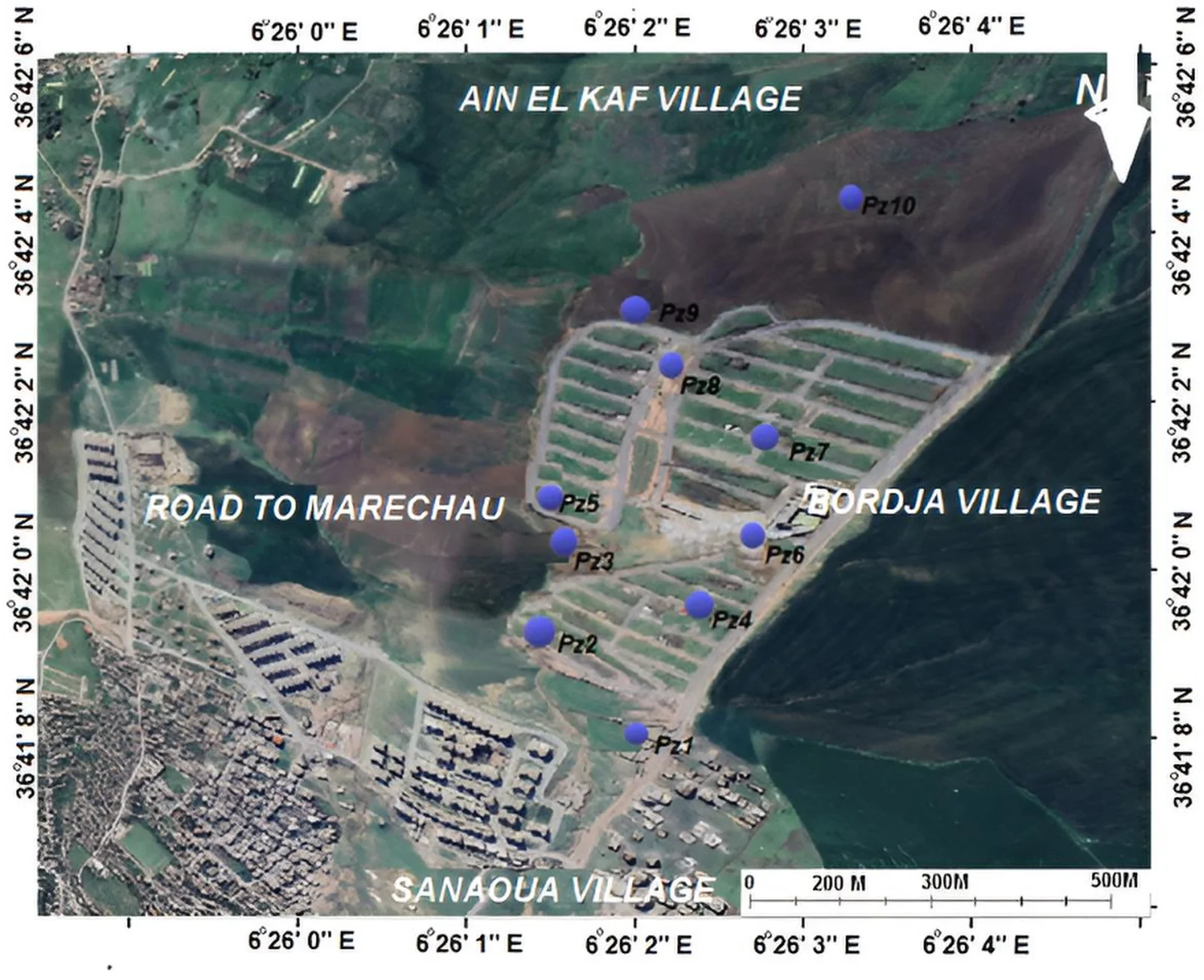
Location map of Piezometers (Pz) in Sanaoua area. Base map was generated using Sentinel-2 imagery (Copernicus Programme, ESA) and Landsat data (USGS). Image processing and map production were carried out using ArcGIS (version 10.0.0.2414, https://www.esri.com).

The locations of the piezometers (Pz) within the study area are shown in Fig. 13. This Figure illustrating the spatial distribution of monitoring points used to evaluate groundwater presence, depth, and temporal variations across the site.

Piezometric monitoring conducted at ten locations over a three-month autumn period (Fig. 13) did not reveal the presence of permanent groundwater within the investigated depth range, apart from short-lived film-type water stagnations. No springs or groundwater resurgences were observed within the site boundaries during the monitoring period. These findings indicate that erosion and slope instability in the study area are primarily driven by surface runoff and episodic infiltration pulses rather than by sustained shallow groundwater pressures. This interpretation is consistent with the findings of Khellaf et al.^5^, who showed that the Mila region is characterized by low-permeability clayey formations, high seasonal rainfall, dominant surface runoff, and limited recharge of the superficial aquifer. Their study also highlighted that groundwater fluctuations and transient infiltration reduce shear strength, increase deformability, and promote shrink–swell behavior, particularly on sloping terrains. However, transient perched water may still develop during intense rainfall events, particularly along thalweg infill zones, at permeability contrasts, or behind cut slopes, and may play a critical role in triggering localized instability. Accordingly, drainage design should prioritize the interception and controlled conveyance of surface runoff, the dissipation of flow energy, and the prevention of localized ponding and infiltration into geotechnically sensitive cut faces.

Runoff concentration in concave hillslopes naturally promotes flow convergence. This increases hydraulic loading along thalwegs and micro-depressions, making them preferential pathways for episodic infiltration rather than uniform recharge flow is intercepted, permeability contrasts are exposed, and lateral subsurface flow is forced upward or slowed. This can easily generate localized saturation zones without requiring a regional aquifer the ERT low-resistivity pockets as: episodically wetted clay lenses, or thalweg infill (colluvium/alluvium with higher moisture retention), is more plausible than a continuous aquifer, especially if: the anomalies are spatially discontinuous, they vary between surveys (if time-lapse exists), or they align with surface drainage geometry. A true aquifer would typically show:

1. more lateral continuity, a clearer structural or stratigraphic control, and less dependence on short-term hydrologic events.
2. Correlating ERT anomalies with recent rainfall history (to confirm transience), if low-resistivity zones align with topographic flow accumulation indices.

### Laboratory characterizations

The physico-mechanical properties of the investigated soils are summarized in Tables 4, 5, 6, 7 and 8: Table 4 presents the chemical analysis, indicating generally low and discontinuous gypsum content. Table 5 shows high water content and degrees of saturation exceeding 90%, confirming near-saturated conditions. Tables 6 and 7 highlight moderate densities and high plasticity indices, classifying the soils as high-plasticity clays. Table 8 summarizes the shear strength and compressibility parameters, revealing low cohesion and moderate compressibility, which explain the observed settlement behavior and instability risks.

**Table 4:** Chimical analyzes of some studied site points.

CD	Sampling depth (m)	CaSO₄ 2H₂O (mg/kg)
2	2.10 to 2.50	17.51
	4.60 to 4.90	8.29
7	2.50 to 2.70	4.06
	4.60 to 4.80	2.40
9	2.40 to 2.60	2.58
	4.75 to 5.00	3.69
12	2.55 to 3.00	Traces
	4.15 to 4.50	Traces
14	2.60 to 3.00	2.03
	3.60 to 4.00	2.02
11	2.50 to 2.70	1.47
	3.70 to 4.00	1.65

**Table 5:** Variation of water content (w) and saturation degres Sr in studied site.

CD	Sampling depth (m)	w (%)	Sr (%)
4	4.60 to 5.00	24.04	99.32
	7.00 to 7.30	24.71	99.97
14	2.60 to 3.00	29.85	99.45
	5.00 to 5.40	24.43	99.94
12	2.55 to 3.00	24.07	95.31
	5.70 to 5.90	23.18	96.85
11	2.50 to 2.70	23.46	98.65
	5.40 to 5.60	24.65	99.88
1	2.20 to 2.50	25.47	88.99
	6.40 to 6.70	22.03	99.86
7	2.50 to 2.70	18.80	86.61
	6.40 to 6.60	19.45	99.67

**Table 6:** Variation of wet density (γ_h_) and dry density (γ_d_) in studied site.

CD	Sampling depth (m)	γ_h_(t/m^3^)	γ_d_(t/m^3^)
11	2.50 to 2.70	1.63	2.01
	5.00 to 5.40	1.61	2.00
2	2.10 to 2.50	148	1.88
	5.70 to 5.90	1.62	2.00
1	2.20 to 2.50	1.51	1.89
	5.60 to 5.80	1.74	2.08
8	2.60 to 2.80	1.72	2.05
	6.50 to 6.70	1.77	2.10
5	2.40 to 2.70	1.67	2.01
	5.40 to 5.60	1.60	2.00
7	2.50 to 2.70	1.68	2.02
	4.60 to 4.90	1.51	1.94

**Table 7:** Variation of Atterberg limits (Wl et Ip) in studied site.

CD	Sampling depth (m)	Wl (%)	Pi (%)
1	2.20 to 2.50	71.86	35.10
	6.40 to 6.70	70.33	38.17
5	2.40 to 2.70	69.79	41.96
	4.70 to 5.00	79.46	49.44
10	2.60 to 2.80	66.38	32.91
	5.60 to 5.80	68.66	23.31
11	2.50 to 2.70	69.65	39.72
	5.40 to 5.60	65.56	37.49

**Table 8:** Characteristics of the Triaxial and odometric compressibility tests of some core drilling sample.

		Triaxial test UU		Œdometric test		
CD	Sampling depth (m)	C (MPa)	φ (°)	σ_c_ (MPa)	Cc (%)	Cg (%)
1	2.00 to 3.00	0.020	16.42	0.147	0.21	0.09
	3.00 to 4.00	0.034	10.57	–	–	–
2	2.00 to 3.00	0.040	09.40	0.12	0.23	0.074
	6.00 to 700	0.038	11.31	–	–	–
4	4.00 to 4.50	0.030	09.59	0.134	0.24	0.07
7	3.00 to 4.00	0.033	12.28	–	–	–
9	5.00 to 6.00	0.032	10.82	0.174	0.32	0.092
12	4.00 to 5.00	0.039	11.24	0.274	0.18	0.066

Gypsum contents range from trace amounts to 24.33 mg/kg, with higher concentrations observed at depths of 2.1–2.7 m (SC1: 16.95 mg/kg; SC2: 17.51 mg/kg; SC5: 24.33 mg/kg) and only trace amounts (< 4 mg/kg) recorded at depths of 3.6–5.0 m (Table 4). According to^70,71^, this distribution indicates a heterogeneous but discontinuous occurrence of gypsum within the soil profile. The soils are characterized by natural water contents ranging from 15.50 to 29.85%, degrees of saturation generally exceeding 90% (locally reaching approximately 99%) (Table 5), wet unit weights of 1.48–1.83 t/m^3^, and dry unit weights of 1.88–2.11 t/m^3^ (Table 6). High Atterberg limits (liquid limit Wl = 61.22–79.46% and plasticity index Pi = 23.31–49.44%) are also observed (Table 7). These parameters, determined in accordance with^31–37,72–75^, − 2, − 12, and − 13, classify the materials as high-plasticity clays (CH) under^76,77^. The soils are nearly saturated and predominantly undrained under in situ conditions.

According to^78^ and ACI 318, the measured gypsum contents correspond to low sulfate exposure classes, indicating limited chemical aggressivity toward concrete. However, ISSMGE soil behavior concepts, together with Eurocode 7^38,39^, indicate that the combination of high saturation, moderate densities, and high plasticity results in high compressibility, low shear strength, and significant shrink–swell potential. These characteristics provide a geotechnical explanation for the observed settlement sensitivity and slope instability in the study area. This interpretation is consistent with recent studies highlighting that soil deformation and settlement in heterogeneous environments are primarily controlled by hydro-mechanical processes and stratigraphic variability. In particular, weak layers embedded within more competent materials govern differential settlement and localized deformation, leading to non-uniform strain distribution and progressive damage^79^. Furthermore, settlement evolution in clay-rich environments typically follows a multi-stage process involving initial rapid deformation, followed by consolidation and long-term creep, with strong spatial variability controlled by moisture conditions and subsurface heterogeneity^80^. In addition, geomorphological controls such as slope morphology, drainage concentration, and lithological contrasts play a fundamental role in localizing instability processes, particularly in fine-grained, moisture-sensitive soils^81^. These combined findings reinforce the interpretation that the Sanaoua site is highly susceptible to differential settlement and slope instability due to the coupled effects of soil plasticity, near-saturated conditions, and geomorphological controls.

Cohesion values ranging from 0.018 to 0.043 MPa, friction angles between 9.4 and 16.4°, compression indices (Cc) of 0.18–0.32, swelling indices (Cg) of 0.049–0.092, and preconsolidation stresses (σ_c_) of 0.12–0.274 MPa (Table 8) characterize the investigated soils. These parameters were derived from unconsolidated–undrained triaxial and oedometer tests conducted in accordance with^25,82–86^. According to the classification and design criteria of Eurocode 7^38,39^, and the Terzaghi–Peck–Mesri framework, these parameters are indicative of soft to medium-stiff cohesive soils exhibiting moderate to high compressibility and normally consolidated to lightly overconsolidated behavior. The combination of low shear strength and high compressibility accounts for the pronounced settlement potential and high sensitivity to undrained and seismic loading. This behavior is consistent with recent studies showing that deformation and instability in weak and heterogeneous geomaterials are governed by progressive damage mechanisms involving cohesion degradation and friction mobilization, which significantly influence stress redistribution, plastic zone development, and post-yield behavior in geotechnical systems^87^. In agreement with Eurocode 7, ISSMGE recommendations, and international seismic design practice, these characteristics indicate that conventional shallow foundations on sloping terrain are generally unsuitable. Accordingly, mitigation strategies should include ground-improvement measures, deep foundation systems, controlled drainage, and conservative deformation criteria to reduce the risk of instability and differential settlement.

The laboratory results indicate that, although gypsum contents are low and discontinuous and therefore pose limited chemical aggressivity to buried concrete, the mechanical and hydro-mechanical behavior of the soils constitutes the principal engineering constraint at the Sanaoua site. The soils are dominated by nearly saturated, high-plasticity clays (CH) characterized by low undrained shear strength, moderate to high compressibility, and normally consolidated to lightly overconsolidated behavior. These properties render the deposits highly sensitive to structural loading, rainfall infiltration, and seismic excitation. The absence of a permanent groundwater does not significantly mitigate this risk, as near-saturated conditions are maintained by transient infiltration and surface-runoff concentration, particularly along thalwegs on stemep slopes. As a result, the soils exhibit low bearing capacity, high settlement potential, pronounced shrink–swell behavior, and increased susceptibility to slope instability and earthquake-induced deformation. Under these conditions, conventional shallow foundations are unsuitable across much of the site. Accordingly, safe and sustainable urban development at Sanaoua requires the implementation of deep or ground-improved foundation systems, rigorous surface and subsurface drainage control, conservative undrained and seismic design assumptions, and careful geotechnical zoning to limit cut-and-fill operations and differential settlement. These measures are fully consistent with the requirements of Eurocode 7 and established international geotechnical practice.

The laboratory, in situ, and geophysical results obtained at the Sanaoua site converge toward a coherent geotechnical model that is fully consistent with the geomorphological, geological, and seismic context of the area. The soils are dominated by nearly saturated, high-plasticity clays exhibiting moderate to high compressibility and low undrained shear strength, which explains the pronounced susceptibility to settlement, deformation, and landsliding observed on this steep, concave hillslope affected by intense erosion and surface-runoff concentration. These laboratory-derived characteristics correlate closely with pressuremeter results, which indicate very low stiffness and bearing capacity within the upper 2 m and a progressive improvement in mechanical properties below depths of approximately 4–6 m. They are also consistent with ERT profiles that image thick, laterally extensive low-resistivity clay-dominated domains (engineering unit U2), locally interrupted by discontinuous resistive carbonate bodies (unit U3). The absence of a permanent groundwater, combined with high degrees of saturation and localized conductive zones identified by ERT, indicates that transient infiltration and surface-water runoff, rather than sustained groundwater pressures, represent the primary triggering mechanisms for instability. These effects are likely to be exacerbated under seismic loading, as demonstrated by recent earthquake-induced landslides in the Mila region. Minor and discontinuous gypsum contents imply limited chemical aggressivity and do not significantly improve the mechanical behavior of the deposits. Overall, the integrated dataset confirms that the Sanaoua site is mechanically stratified and laterally heterogeneous, with compressible clay layers dominating most foundation levels. Under conditions of steep slopes and significant seismic hazard, safe urban development therefore requires depth-controlled foundation solutions, rigorous surface- and subsurface-drainage management, and careful geotechnical zoning to mitigate the risks of differential settlement and slope instability.

#### Bearing capacity (q_ad_) and settlement (Δ***H***) assessment

**Fig. 14:**
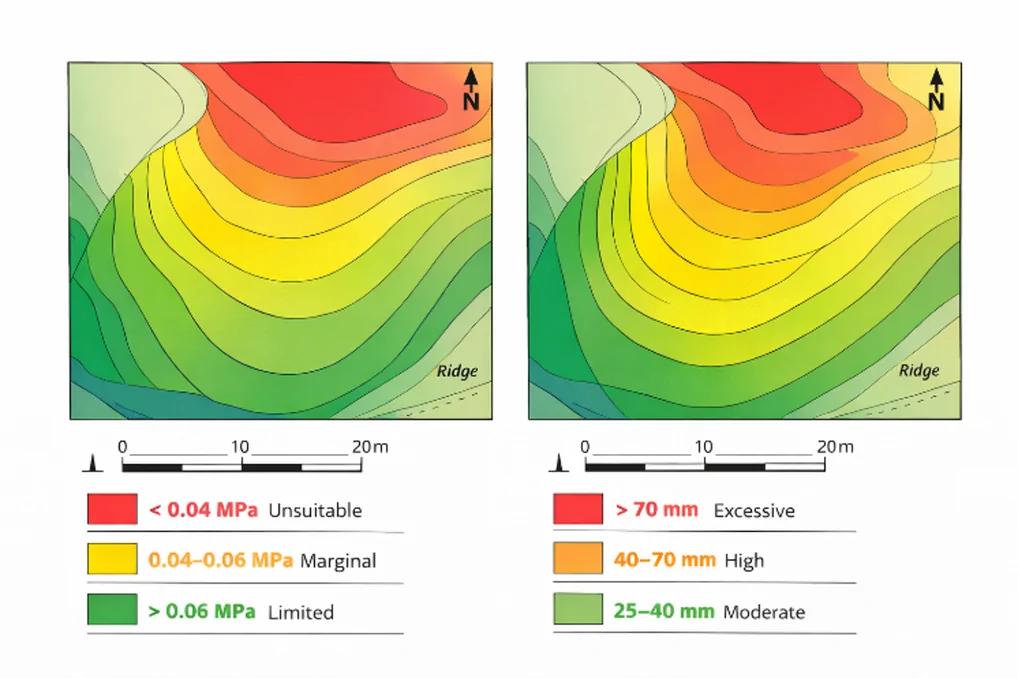
Geotechnical zoning map based on estimated admissible bearing capacity and settlement behavior. Base map was generated using Sentinel-2 imagery (Copernicus Programme, ESA) and Landsat data (USGS). Image processing and map production were carried out using QGIS (version 3.16 https://qgis.org).

Based on the laboratory results and in accordance with Eurocode 7^38,39^, and classical geotechnical theory, the admissible bearing capacity (q_ad_) and settlement (Δ*H*) results were spatially interpolated to produce the maps shown in Fig. 14. This Figure illustrating ground suitability for construction. Low bearing capacity (< 0.04 MPa) and high settlement (> 70 mm) define unsuitable zones, while moderate to high capacity and reduced settlement identify areas with improved geotechnical conditions.

The spatial distribution of admissible bearing capacity (q_ad_) and settlement (Δ*H*) shown in Fig. 14 reveals a clear geotechnical zoning of the Sanaoua site, primarily controlled by soil compressibility, degree of saturation, and the depth to mechanically stiffer horizons. Areas characterized by low q_ad_ values correspond to highly plastic, nearly saturated clayey soils with low undrained shear strength, for which laboratory results indicate high compressibility. These zones systematically coincide with the highest predicted settlement values, commonly exceeding serviceability limits for conventional shallow foundations as defined by Eurocode 7^39^.

Intermediate zones display moderate bearing capacity and settlement values, reflecting partial improvement in soil density and stiffness with depth, but still requiring cautious foundation design and strict deformation control. In contrast, sectors exhibiting relatively higher bearing capacity are associated with shallower occurrences of stiffer clay layers or localized competent horizons, resulting in reduced settlement magnitudes and more favourable load-transfer conditions. Overall, the interpolated maps demonstrate that settlement, rather than shear failure, governs foundation performance across most of the site. This interpretation is consistent with recent studies emphasizing that bearing-capacity degradation and settlement are strongly governed by hydro-mechanical coupling, effective-stress reduction, and spatial variability of ground conditions, and that reliable prediction of deformation requires integration of field observations with advanced analytical or data-driven approaches^88^. It is also in agreement with recent experimental work showing that load-settlement behaviour and ultimate bearing response are highly sensitive to subsurface discontinuities and heterogeneity, which can significantly modify failure mechanisms and differential deformation patterns^89^. The zoning results therefore provide a practical basis for geotechnical zoning, foundation-type selection, and the identification of areas where ground-improvement measures or deep foundation systems are required to ensure safe and sustainable urban development.

### Limitations of the study

Despite the comprehensive multi-method investigation conducted in this study, several limitations should be acknowledged. First, the depth of exploration was limited to approximately 10 m, which may not fully capture deeper geological conditions or the variability of the bedrock interface across the entire site. Second, the number and spatial distribution of in situ tests (core drilling, pressuremeter, dynamic penetration, and ERT profiles) provide a representative but still discrete coverage of the study area, which may not fully resolve small-scale heterogeneities inherent to clayey deposits containing limestone blocks.

Third, the piezometric monitoring was conducted over a relatively short period (3 months during the autumn season), and therefore may not reflect long-term groundwater fluctuations or extreme hydrological conditions. In addition, the interpretation of geophysical data (ERT) remains indirect and relies on correlations with borehole data, which introduces a degree of uncertainty in defining subsurface boundaries.

Finally, the proposed geotechnical model is based on the Mohr–Coulomb framework, which, although widely used in engineering practice, simplifies the complex behavior of highly plastic and partially saturated soils, particularly under cyclic or seismic loading conditions.

Future work should therefore include deeper investigations, longer-term hydrogeological monitoring, and advanced numerical modeling approaches to better capture the coupled hydro-mechanical behavior of these deposits.

## Correlation of datasets and development of the geotechnical engineering model (mohr–coulomb)

Given the complexity of the Sanaoua subsurface, a geotechnical model capable of synthesizing geological, geomorphological, hydrogeological, and mechanical processes is required. Accordingly, the Mohr–Coulomb constitutive model was adopted as an integrative geoenvironmental framework to represent the overall mechanical response of the ground, rather than as a purely structural design formulation.

### Correlations of data

The integrated analysis of in situ test results, geophysical data, laboratory parameters, and hydrogeological observations provides a coherent representation of subsurface conditions, indicating that the following characteristics can be distinguished (Table 9). The Table 9 summarizes the correlation between observed geotechnical features and measurements at the Sanaoua site. This table shows a clear progression from very soft surficial material to highly competent limestone, guiding foundation design, stability assessment, and excavation planning.

**Table 9:** Summary of geotechnical parameter–condition correlations at the Sanaoua site.

Observed feature	CD/Lab	Ps	DPT	ERT	Engineering meaning
Thin surface layer (0–2 m)	Colluvium + altered clay, high Wl–Ip, Sr ≈ 90–99%	Very low E_m_ (≈ 6 MPa), low P_*l*_ (≈ 0.45 MPa)	Low Pr(min)	Moderate–low ρ	Highly deformable, erosion-prone, unsuitable for foundations
Main clay mass (≈ 2–6 m)	CH clays, Cᵤ ≈ 0.018–0.043 MPa, Cc ≈ 0.18–0.32	E_m_ ↑ (15–35 MPa), P_*l*_ ≈ 0.8–1.5 MPa	Moderate Pr	Low ρ (10–40 Ω.m)	Governing layer for Δ*H* & undrained stability
Blocky clay/marl (≈ 6–10 m)	Clay with limestone blocks	E_m_ 35–60 MPa, P_*l*_ 1.5–2.8 MPa	High Pr(max), partial refusal	Intermediate–high ρ	Transition to competent horizon
Local limestone bodies	Limestone blocks	Very high P_*l*_ & E_m_ locally	BR/N 50	High ρ (150 Ω.m)	Controls q_ad_ heterogeneity

### Practical engineering model for the sanaoua site

#### Purpose and philosophy of the model

The Sanaoua site is characterized by a complex interaction between stratigraphy, geomorphology, hydrogeology, and soil mechanical behavior. The objective of the proposed engineering model is not to idealize the ground as a homogeneous medium, but rather to capture the dominant mechanisms governing settlement, stability, and bearing performance under static, hydrological, and seismic loading conditions. Consequently, the model emphasizes mechanical behavior and process control—such as settlement response, pore-pressure sensitivity, and material heterogeneity—rather than relying solely on lithological classification.

Accordingly, the site is represented by a layered, depth-dependent engineering model in which each layer is assigned specific constitutive parameters, deformation characteristics, and design implications. The Mohr–Coulomb framework is adopted as a pragmatic and widely accepted engineering representation, suitable for limit-equilibrium and finite-element analyses, provided that parameter selection is conservative and explicitly linked to drainage and loading conditions.

#### Engineering soil units and mechanical roles

Based on the integrated interpretation of core drilling data, laboratory test results, pressuremeter measurements, dynamic penetration resistance, and ERT profiles, the subsurface of the Sanaoua site is subdivided into three practical engineering soil units (Table 10). Table 10 summarizes the main engineering soil units identified at the Sanaoua site, their depth ranges, lithological characteristics, and key mechanical behaviors. Overall, the table highlights the transition from highly deformable, near-surface soils to more competent, load-bearing materials at depth. It provides essential guidance for foundation selection, stability assessment, and construction planning.

**Table 10:** Engineering soil units and associated mechanical behavior at the Sanaoua site.

Engineering unit	Typical depth range (m)	Lithological description	Key geotechnical characteristics	Mechanical behavior	Engineering implications
U1	0–1/2	Shallow colluvial and remolded clay	Very low stiffness; high plasticity; strong moisture sensitivity; very low Ménard modulus and limit pressure; high water content and plasticity index	Negligible bearing capacity; highly deformable; prone to erosion and shallow instability	Unsuitable for foundations; responsible for surface erosion, shallow slides, and construction-stage instability; should be removed, bypassed, or structurally bridged
U2	~ 2 to 5–7	Thick, nearly saturated high-plasticity clay (CH)	Low undrained shear strength; normally consolidated to lightly overconsolidated state; moderate to high compressibility; moderate stiffness increasing with depth but insufficient to control settlement	Governs settlement, undrained stability, and seismic response; deformation-controlled behavior	Shallow foundations generally unsuitable; controls most design decisions; requires deep foundations, ground improvement, and conservative undrained and seismic design assumptions
U3	~ 6 to 12 (variable)	Stiff clay with discontinuous limestone blocks and locally weathered carbonate material	Higher stiffness and limit pressures; high DPT peak resistance; resistive ERT anomalies; heterogeneous structure	Most mechanically competent unit but laterally discontinuous; behaves as a soil–block matrix rather than continuous bedrock	Preferred load-bearing unit for piles or micropiles; local verification required to limit differential settlement due to heterogeneity

#### Hydro-mechanical behavior and drainage conditions

Piezometric monitoring indicates the absence of a permanent groundwater within the investigated depth range. Nevertheless, laboratory results reveal degrees of saturation close to 100%, and ERT profiles identify conductive zones associated with transient moisture accumulation. Together, these observations demonstrate that the hydro-mechanical behavior of the site is governed by transient perched water conditions generated by rainfall infiltration, surface-runoff concentration along thalwegs, and local permeability contrasts.

From an engineering standpoint, these conditions imply that short-term loading should be treated as undrained, particularly within the dominant clay unit (U2), whereas long-term behavior must account for slow consolidation and creep processes. Consequently, slope instability and deformation are more likely to be triggered by intense rainfall events and seismic shaking than by sustained groundwater pressures.

### Constitutive representation and design parameters

Within the proposed engineering model, the Mohr–Coulomb constitutive law is implemented using layer-specific parameters and an explicit distinction between short-term (undrained) and long-term (drained) conditions. For engineering unit U2, conservative design assumptions require the adoption of a friction angle close to zero under undrained conditions, with stability governed primarily by undrained cohesion. Under long-term conditions, low effective cohesion combined with moderate friction angles is considered, consistent with the high plasticity and mineralogical characteristics of the clayey materials.

Soil stiffness is represented using pressuremeter-derived elastic moduli for short-term deformation analysis and oedometer-derived moduli for settlement calculations. The model explicitly acknowledges that settlement, rather than bearing-capacity failure, governs serviceability performance across most of the site.

#### Foundation behavior and constructability

The proposed engineering model clearly indicates that conventional shallow foundations founded within engineering units U1 or U2 are unsuitable because of excessive settlement potential and low undrained shear strength. Raft foundations may be considered only in limited areas where unit U3 occurs at shallow depth and where appropriate ground-improvement and drainage measures are implemented. In most cases, safe and reliable load transfer requires the use of deep foundation systems (micropiles or piles) anchored within the stiffer U3 unit, with explicit consideration of spatial variability in toe resistance.

Differential settlement represents a major design risk owing to the lateral discontinuity of mechanically competent horizons. Consequently, foundation systems must be designed to accommodate this variability, either through structural continuity or through adaptable foundation layouts capable of redistributing loads without inducing excessive deformation.

#### Slope stability and seismic response

Slope stability at the Sanaoua site is primarily governed by the behavior of the saturated clay unit (U2) under undrained conditions. Rainfall-induced pore-pressure increases and seismic loading can rapidly reduce effective stress, leading to shallow to intermediate-depth slope failures. Accordingly, the practical engineering model requires that slope-stability analyses be conducted using undrained shear-strength parameters for short-term conditions, with the inclusion of pseudo-static seismic loading consistent with the regional seismic hazard.

Surface-water management represents a key mitigation measure. The model highlights that effective control of runoff and limitation of infiltration are more efficient than deep drainage systems, given the absence of a permanent groundwater. Proper surface-water interception and conveyance are therefore essential for reducing instability risks at the site.

#### Integrated practical model (design synthesis)

In summary, the Sanaoua site is best represented by a layered, hydro-mechanically sensitive engineering model in which a thick, nearly saturated clay unit governs settlement behavior, slope stability, and seismic response, while deeper, heterogeneous stiff layers provide localized bearing support. The proposed engineering model integrates stratigraphic, mechanical, and hydrological controls into a coherent framework that is well suited for foundation design, slope-stability assessment, and urban development planning in a geomorphologically complex and seismically active environment.

## Conclusion

This study presents an integrated geotechnical, geophysical, and hydrogeological investigation of the Sanaoua sector in the Mila Basin (northeastern Algeria), aimed at assessing subsurface conditions and their implications for sustainable urban development. The combined analysis of core drilling, pressuremeter testing, dynamic penetration tests, electrical resistivity tomography (ERT), and piezometric monitoring provides a coherent understanding of the stratigraphic architecture and mechanical behavior of the site.

The results highlight a predominantly clay-dominated subsurface, characterized by thick, high-plasticity and near-saturated deposits containing gypsum fragments and limestone blocks. These materials exhibit low shear strength, high compressibility, and significant sensitivity to water-content variations. Pressuremeter and penetration test results demonstrate a clear depth-dependent improvement in mechanical properties, with very low stiffness and bearing capacity in the upper layers and more competent conditions generally reached at depths exceeding 6–8 m. Settlement analyses confirm that shallow foundations are associated with excessive deformation, whereas deeper horizons provide more favorable support conditions.

Geophysical investigations further reveal strong lateral heterogeneity, with conductive clay-rich domains locally interrupted by resistive carbonate bodies. This heterogeneity explains the pronounced spatial variability in bearing capacity and settlement behavior observed across the site. Piezometric monitoring indicates the absence of a permanent groundwater table within the investigated depth, suggesting that slope instability and deformation processes are primarily controlled by surface runoff and transient perched water conditions rather than sustained groundwater pressures.

Based on these findings, a simplified engineering–geological model was developed within a Mohr–Coulomb framework to support practical design applications. The results indicate that shallow foundations are generally unsuitable in most parts of the site, and that deep foundations or ground-improvement techniques, combined with effective surface-water management, are required to mitigate settlement and instability risks. The study also emphasizes the need for geotechnical zoning and adaptable foundation solutions to account for the strong lateral variability of subsurface conditions.

Despite the comprehensive multi-method approach, several limitations should be acknowledged. The depth of investigation was limited to approximately 10 m and may not fully capture deeper geological variability. The spatial distribution of in situ tests, although representative, remains discrete and may not resolve all small-scale heterogeneities. In addition, piezometric monitoring was conducted over a relatively short period and may not reflect long-term hydrogeological behavior or extreme climatic events. The interpretation of geophysical data involves inherent uncertainties, and the use of the Mohr–Coulomb model simplifies the complex behavior of highly plastic and partially saturated soils.

Future research should therefore focus on deeper and more extensive site investigations, longer-term hydrogeological monitoring, and the application of advanced numerical modeling approaches that better capture coupled hydro-mechanical and seismic effects. Further work on ground-improvement techniques and optimized foundation design strategies is also recommended to support safe and sustainable urban development in similar geological environments.

## Data Availability

The datasets used and/or analyzed during the current study are available from Prof. Abdelaziz Rabehi (Abdelaziz.rabehi@univ-djelfa.dz) on reasonable request.
